# Adverse events associated with anti-IL-17 agents for psoriasis and psoriatic arthritis: a systematic scoping review

**DOI:** 10.3389/fimmu.2023.993057

**Published:** 2023-01-31

**Authors:** Jiao Wang, Chunxiao Wang, Liu Liu, Seokgyeong Hong, Yi Ru, Xiaoying Sun, Jiale Chen, Miao Zhang, Naixuan Lin, Bin Li, Xin Li

**Affiliations:** ^1^ Department of Dermatology, Yueyang Hospital of Integrated Traditional Chinese and Western Medicine, Shanghai University of Traditional Chinese Medicine, Shanghai, China; ^2^ Institute of Dermatology, Shanghai Academy of Traditional Chinese Medicine, Shanghai, China; ^3^ Department of Dermatology, Shanghai Skin Disease Hospital, Shanghai, China

**Keywords:** anti-IL-17, biological agents, adverse events, meta-analysis, systematic review

## Abstract

**Background:**

Anti-interleukin (IL)-17 biological agents (BAs) have significant efficacy in the treatment of psoriasis and psoriatic arthritis; however, adverse events (AEs) are common, and their safety has not been systematically evaluated.

**Objectives:**

The purpose of this systematic review and meta-analysis was to summarize the number and corresponding rates of AEs caused by anti-IL-17 BAs in patients with psoriasis and psoriatic arthritis to improve clinical decision-making regarding their use.

**Methods:**

PubMed, Embase, Cochrane Library, and Web of Science databases were independently searched by three authors for articles on the treatment of psoriasis with anti-IL-17 BAs that were published before March 1, 2022, and included at least one AE. Dichotomous variables and 95% confidence intervals (CI) were analyzed using R software (version 4.1.3) and the Meta and Metafor software packages. Funnel plots and meta-regression were used to test for the risk of bias, *I^2^* was used to assess the magnitude of heterogeneity, and subgroup analysis was used to reduce heterogeneity.

**Results:**

A total of 57 studies involving 28,424 patients with psoriasis treated with anti-IL-17 BAs were included in the meta-analysis. Subgroup analysis showed that anti-IL-17A (73.48%) and anti-IL-17A/F (73.12%) BAs were more likely to cause AEs than anti-IL-17R BAs (65.66%). The incidence of AEs was as high as 72.70% with treatment durations longer than one year, and long-term use of medication had the potential to lead to mental disorders. Infection (33.16%), nasopharyngitis (13.74%), and injection site reactions (8.28%) were the most common AEs. Anti-IL-17 BAs were most likely to cause type α (33.52%) AEs. Type δ AEs (1.01%) were rarely observed.

**Conclusions:**

Anti-IL-17 BAs used for the treatment of psoriasis and psoriatic arthritis caused a series of AEs, but the symptoms were generally mild.

## 1 Introduction

Psoriasis has long been a significant research topic in dermatology. Psoriasis usually affects the head, trunk, and elbows. Thimble nails and wispy hair are idiopathic symptoms. Psoriasis is known as an “undying disease” due to recurring outbreaks that are difficult to cure and can lead to depression and even suicidal tendencies in severe cases (1). Unfortunately, the incidence of psoriasis has increased annually. Psoriasis can occur with a variety of comorbidities such as cardiovascular disease, chronic obstructive pulmonary disease, metabolic disorders, and psoriatic arthritis (PSA); approximately 30% of patients with psoriasis develop PSA (2). There is no one-stop solution for the treatment of moderate-to-severe psoriasis. Dermatologists commonly use glucocorticoids, vitamin D analogs, calcium-regulated phosphatase inhibitors, and phototherapy to control the course of psoriasis, but the results are unsatisfactory.

**Figure 1:**
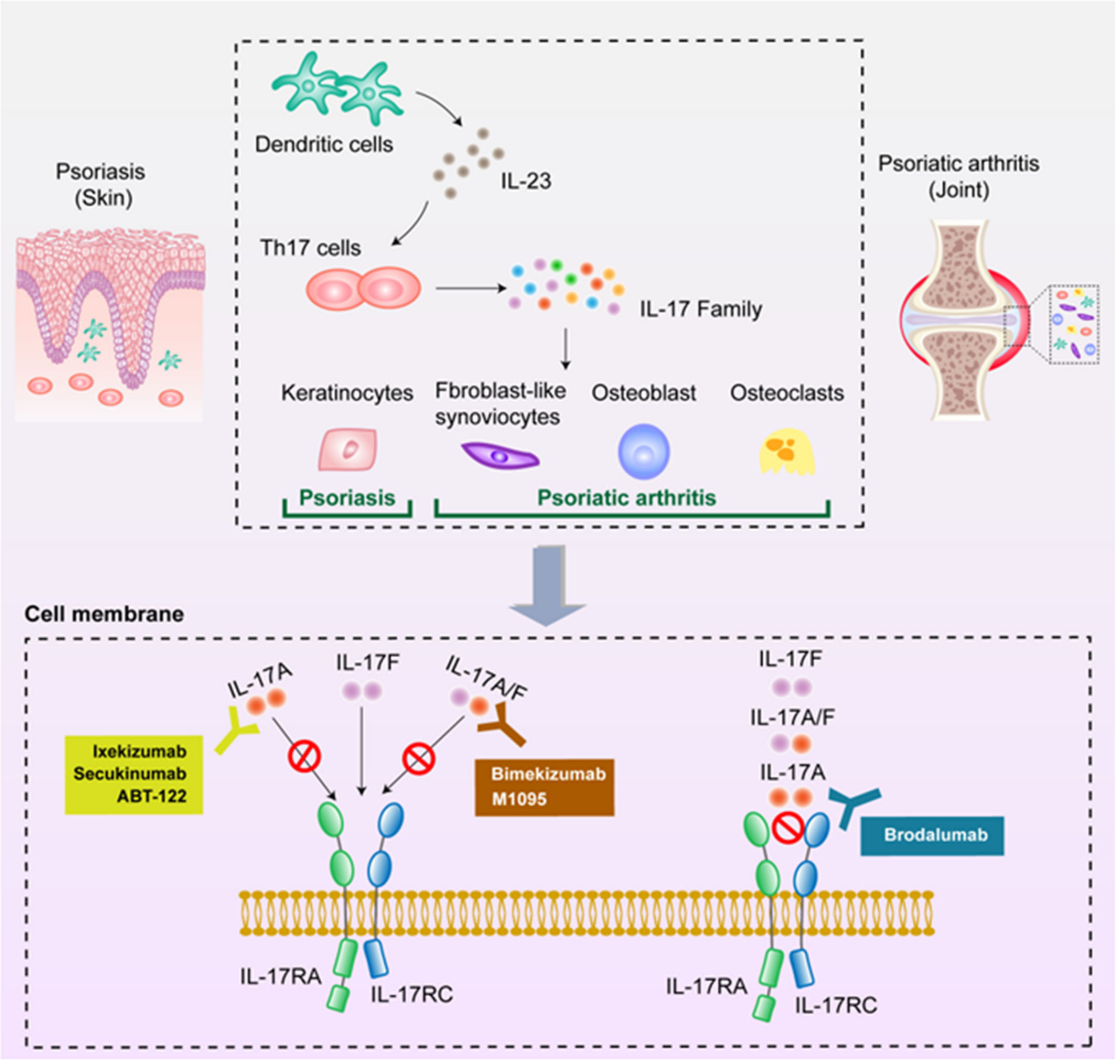
Schematic diagram of the mechanism of action of anti-interleukin-(IL)-17 drugs. The mechanism of anti-IL-17 monoclonal antibodies in psoriasis and psoriatic arthritis. The pathogenesis of both psoriasis and psoriatic arthritis is closely related to the IL-23/IL-17 axis. First, dendritic cells secrete IL-23 to activate Th17 cells and then the activated Th17 cells secrete large amounts of IL-17, thereby triggering an inflammatory cascade. Next, the IL-17 family induces excessive proliferation of keratinocytes, resulting in localized papules, erythema, and silvery-white plaques on the skin. In addition, the IL-17 family act on the joint to activate synovial fibroblasts, osteoblasts, and osteoclasts, triggering local tissue inflammation. This study included three types of monoclonal antibodies that alleviate psoriasis and psoriatic arthritis by blocking IL-17A, IL-17R, and IL-17A/F, thereby interrupting the inflammatory cascade.

The IL-23/Th17 immune axis is thought to play a central role in the development of psoriasis. Th17-related cytokines, such as interleukin (IL)-17A and IL-17F, are significantly elevated in psoriasis. Previous studies have shown that the expression levels of IL-17A and IL-17F are up to eight times higher in psoriatic skin lesions than those in healthy patients. IL-17 causes increased proliferation of keratinocytes and inflammation by stimulating pro-inflammatory cytokines, pro-proliferative cytokines, antimicrobial peptides, and chemokines. IL-17 can also upregulate inflammatory factors such as IL-6 and intracellular adhesion molecule-1 in endothelial cells, further enhancing the inflammatory response. Similarly, IL-17A plays a central role in PSA by enhancing the inflammatory response and promoting joint damage. IL-17A targets osteoblasts, osteoclast precursors, and synovial-like joint fibroblasts (3) (**Figure 1**). Moreover, clinical studies have shown that the discontinuation rate of traditional systemic therapy is as high as 66% owing to its low efficacy and side effects (4).

BAs are highly active immunologic agents that exert their effects by blocking specific inflammatory factors produced by the immune system. BAs are commonly used in the treatment of autoimmune diseases such as rheumatoid arthritis, PSA, systemic lupus erythematosus, and ankylosing spondylitis (5). In 2004, the European Medicines Agency (EMA) approved the first BA, etanercept; adalimumab and ustekinumab have since emerged (6). A meta-analysis of 28 studies involving IL-12, IL-23, and IL-17 inhibitors showed these biologics to be more effective than placebos in the short term, although they increased the risk of adverse events (AEs) (7). The primary reason that BAs cause AEs is that they are highly immunogenic proteins, such as monoclonal antibodies, fusion proteins, and cytokines, that directly act on the immune system. Monoclonal antibodies targeting IL-17, IL-17A/F, and IL-17R have been developed and put into clinical use, which has benefited patients with moderate-to-severe psoriasis. Compared to traditional therapies, BAs have a high curative effect, are simple and convenient to use, and are favored by the majority of patients (8). Secukinumab, a humanized IgG1 monoclonal antibody against IL-17A, was first approved by the FDA in 2015 for the treatment of moderate-to-severe plaque psoriasis, and in 2016 for the treatment of PSA (9). Subsequently, ixekizumab, a humanized IgG4 monoclonal antibody against IL-17A, was developed. The primary mechanism by which IL-17 antibodies attach to cytokines and prevent them from interacting with receptors to block the inflammatory cascade (10). A real-world study monitored 645 psoriasis patients taking ixekizumab and 1152 taking secukinumab for more than a year and showed lower discontinuation rates and higher adherence to ixekizumab (11). Bimekizumab is a humanized IgG1 monoclonal antibody that acts on the dual targets IL-17A and IL-17F. Due to its dual inhibitory effect, bimekizumab more strongly downregulates inflammatory factors, and its clinical efficacy is also considered to be better than that of the anti-IL-17A monoclonal antibody (12). Brodalumab acts on IL-17 receptors on the surface of keratinocytes.

Currently, there is no summary of the safety of IL-17 BAs. To improve their clinical use, it is necessary to comprehensively summarize and analyze the safety of IL-17 BAs. Therefore, the purpose of this systematic review and meta-analysis was to evaluate the incidence of AEs caused by IL-17 BAs to provide data that contribute to better decision-making regarding their clinical application, rational drug use, and reducing the occurrence of AEs.

## 2 Methods

### 2.1 Literature search strategy

We used a combination of subject terms and free words to retrieve relevant articles published before March 1, 2022 from four major databases: PubMed, Embase, Cochrane, and Web of Science. The search terms included “psoriasis;” the IL-17A-related items “ixekizumab,” “ABT-122,” and “secukinumab;” the IL-17A/F related BAs “bimekizumab” and “M1095;” and the IL-17R related biologic “brodalumab.” Search terms for biologics also included trade names. The keywords “safety,” “adverse reactions,” “adverse events,” and “side effects” were also included. Additionally, our search covered anti-IL-17 agents for both psoriatic arthritis and psoriasis. This systematic review was performed in accordance with the Cochrane Handbook for Systematic Reviews of Interventions (**Supplementary Table 1**) (13).

### 2.2 Study selection

The inclusion criteria were as follows: (1) randomized controlled trials using anti-IL-17 biologics and (2) studies containing more than one AE. The exclusion criteria were as follows: (1) non-anti-IL-17 drugs, (2) non-randomized controlled trials, (3) preclinical studies, (4) non-experimental articles such as case reports, reviews, meta-analyses, and conferences, (5) articles with incomplete basic information or that provided the abstract only. Two authors (JW and LL) independently screened the articles, and any difference of opinions was resolved by a third-party (XL) intervention.

### 2.3 Data extraction

Three authors (JW, CW, and LL) independently extracted the following information: (1) first author and publication year; (2) research stage; (3) disease diagnosis; (4) BA type and target, trade name, and constituent; (5) total number of individuals included in the study; (6) experimental dose of monoclonal antibody; (7) mode of administration; and (8) study registration number. AEs-related data were also included. AEs are divided into five categories according to their target and biological consequences: α, β, γ, δ, and ϵ. Type α is often a systemic inflammatory response caused by the release of high levels of pro-inflammatory factors such as IL-6, IL-8, and INF. Type β is often considered to be an immediate or delayed hypersensitivity reaction. Type γ is associated with immune imbalance. Type δ is related to cross-reactions. Type ϵ typically represents psychiatric diseases and neurological symptoms (**Supplementary Table 2**) (14).

### 2.4 Quality assessment

The risk of bias in the included randomized controlled trials (RCTs) was categorized as high, medium, or low, and was assessed using the Cochrane risk of bias tool, which comprises seven parameters. The specific parameters are presented in **Supplementary Table 3**. Independent evaluation was performed by three authors (JW, LL, and CW), and disagreements were resolved by a fourth author (XL).

### 2.5 Statistical analysis

Dichotomous variables and 95% confidence intervals (CI) were analyzed using R software (version 4.1.3) and the Meta and Metafor software packages. The original rates were transformed using the Freeman–Tukey double arcsine transformation and then tested for normality. Metaprop was used to calculate the rates and 95% confidence intervals for each independent study, and the *Q* test and *I^2^* value were used to analyze the heterogeneity among groups. An I^2^ >50% indicated that the heterogeneity was high; in these cases, a subgroup analysis was required, and a random effects model was used.

## 3 Results

### 3.1 Characteristics of the included studies

The final analysis included 57 RCTs; the process used to screen articles is shown in **Supplementary Figure 1**. Adverse reactions caused by at least one monoclonal antibody were recorded from each study; if a study included multiple dose cohorts, each dose was analyzed independently. The characteristics of adverse events caused by anti-IL-17 agents are summarized in **Table 1**.

**Table 1:** Characteristics of the 57 included studies.

Study	Design (Phase)	Diagnosis	Drug/Target	Total number of patients (M/F)	Dose (mg/kg)	mode of administration	Identifier
Blauvelt A 2021 (15)	III	PSO	Ixekizumab/IL-17A	1346 (N/A)	80	i.h.	NCT01646177
Reich K 2019 (16)	III	PSO	Secukinumab/IL-17A	514 (342/172)	300	i.h.	NCT03090100
Mease PJ 2020 (17)	IIIb/IV	PSA	Ixekizumab/IL-17A	566 (312/254)	80	i.h.	NCT03151551
Smolen JS 2020 (18)	IIIb/IV	PSA	Ixekizumab/IL-17A	566 (312/254)	80	i.h.	NCT03151551
Mrowietz U 2019 (19)	IIIb	PSO	Secukinumab/IL-17A	237 (51/186)	300; 150	i.h.	NCT02008890
Chandran V 2020 (20)	III	PSA	Ixekizumab/IL-17A	386 (N/A)	80	N/A	NCT01695239
Gelfand JM 2020 (21)	III	PSO	Secukinumab/IL-17A	91 (61/30)	300	i.h.	NCT02690701
Mease PJ 2017 (22)	III	PSA	Ixekizumab/IL-17A	417 (192/225)	80	i.h.	NCT01695239
McInnes IB 2017 (23)	N/A	PSA	Secukinumab/IL-17A	299 (N/A)	300; 150; 75	N/A	NCT01752634
Bagel J 2017 (24)	IIIb	PSO	Secukinumab/IL-17A	102 (N/A)	300	i.h.	NCT02267135
van der Heijde D 2018 (25)	III	PSA	Ixekizumab/IL-17A	191 (80/111)	80	i.h.	NCT01695239
Okubo Y 2019 (26)	N/A	PSO	Secukinumab/IL-17A	13 (9/4)	300	N/A	NCT01406938
Imafuku S 2017 (27)	III	PSO	Ixekizumab/IL-17A	1296 (N/A)	80	N/A	NCT01474512
Paul C 2015 (28)	III	PSO	Secukinumab/IL-17A	182 (125/57)	300; 150	i.h.	NCT01636687
Blauvelt A 2017 (29)	III	PSO	Ixekizumab/IL-17A	771 (512/259)	80	N/A	NCT01646177
Reich K 2020 (30)	IIIb	PSO	Ixekizumab/IL-17A	54 (42/12)	80	i.h.	NCT02634801
Wu NL 2017 (31)	III	PSO	Secukinumab/IL-17A	51 (41/10)	300; 150	i.h.	N/A
Gottlieb A 2017 (32)	IIIb	PSO	Secukinumab/IL-17A	205 (112/93)	300; 150	i.h.	NCT01806597
Paul C 2019 (33)	IIIb	PSO	Ixekizumab/IL-17A	136 (90/46)	80	i.h.	NCT02561806
Ohtsuki M 2014 (34)	III	PSO	Secukinumab/IL-17A	58 (49/9)	300; 150	i.h.	NCT01365455
Valenzuela F 2017 (35)	III	PSO	Ixekizumab/IL-17A	1604 (1061/543)	80	N/A	NCT01646177
LeoNardi C 2018 (36)	III	PSO	Ixekizumab/IL-17A	1346 (N/A)	80	i.h.	NCT01646177
Reich K 2017 (37)	IIIb	PSO	Ixekizumab/IL-17A	302 (202/100)	80	i.h.	NCT02561806
Sticherling M 2017 (38)	N/A	PSO	Secukinumab/IL-17A	200 (124/76)	150	i.h.	NCT02474082
Blauvelt A 2017 (39)	III	PSO	Ixekizumab/IL-17A	1226 (N/A)	80	i.h.	NCT01474512; NCT01597245
Warren RB 2020 (40)	III	PSO	Secukinumab/IL-17A	327 (213/114)	300	i.h.	NCT03478787
Mease PJ 2018 (41)	II	PSA	ABT-122/IL-17A	240 (121/119)	240; 120	i.h.	NCT02349451
Genovese MC 2018 (42)	III	PSA	Ixekizumab/IL-17A	310 (146/164)	80	i.h.	NCT02349295
Körber A 2018 (43)	III	PSO	Secukinumab/IL-17A	906 (407/498)	300	i.h.	NCT01365455; NCT01358578; NCT02074982
Griffiths CE 2015 (44)	III	PSO	Ixekizumab/IL-17A	2562 (1739/823)	80	i.h.	NCT01597245; NCT01646177
Richard G 2014 (45)	III	PSO	Secukinumab/IL-17A	2044 (1438/606)	300; 150	i.h.	NCT01365455; NCT01358578
Kenneth B 2014 (46)	II	PSO	Ixekizumab/IL-17A	129 (N/A)	10; 25; 75; 150	i.h.	NCT01107457
Blauvelt A 2021 (47)	IV	PSO	Ixekizumab/IL-17A	1025 (N/A)	80	i.h.	NCT03573323
Rich P 2013 (48)	II	PSO	Secukinumab/IL-17A	404 (306/98)	150	i.h.	NCT00941031
Gordon KB 2016 (49)	III	PSO	Ixekizumab/IL-17A	3866 (2622/1244)	80	i.h.	NCT01474512; NCT01597245; NCT01646177
Bagel J 2021 (50)	IIIb	PSO	Secukinumab/IL-17A	1102 (N/A)	300	i.h.	NCT02826603
Lebwohl MG 2020 (51)	N/A	PSO	Ixekizumab/IL-17A	1274 (N/A)	80	N/A	NCT01646177
Stebut EV 2019 (52)	N/A	PSO	Secukinumab/IL-17A	151 (102/49)	300; 150	N/A	N/A
Leonardi C 2020 (53)	III	PSO	Ixekizumab/IL-17A	206 (140/66)	80	N/A	NCT01597245
Thaci D 2015 (54)	IIIb	PSO	Secukinumab/IL-17A	676 (481/195)	300	i.h.	NCT02074982
Nash P 2017 (55)	III	PSA	Ixekizumab/IL-17A	363 (169/194)	80	i.h.	NCT02349295
Mease P 2017 (56)	III	PSA	Secukinumab/IL-17A	996 (500/496)	300; 150	i.h.	NCT02404350
D’Agostino MA 2021 (57)	III	PSA	Secukinumab/IL-17A	166 (75/91)	300; 150	i.h.	NCT02662985
McInnes IB 2020 (58)	III	PSA	Secukinumab/IL-17A	397 (N/A)	150	i.h.	NCT01752634
Mease PJ 2020 (59)	III	PSA	Brodalumab/IL-17R	962 (483/479)	140	i.h.	NCT02029495; NCT02024646
Seo SJ 2020 (60)	III	PSO	Brodalumab/IL-17R	62 (38/24)	210	i.h.	NCT02982005
Nakagawa H 2015 (61)	II	PSO	Brodalumab/IL-17R	151 (120/31)	70; 140; 210	i.h.	N/A
Papp KA 2012 (62)	II	PSO	Brodalumab/IL-17R	198 (127/71)	70; 140; 210; 280	i.h.	NCT00975637
Pinter A 2021 (63)	IV	PSO	Brodalumab/IL-17R	210 (145/65)	210	i.h.	NCT03331835
Reich K 2021 (64)	III	PSO	Bimekizumab/IL-17A/F	567 (406/161)	320	i.h.	NCT03370133
Gordon KB 2021 (65)	III	PSO	Bimekizumab/IL-17A/F	435 (313/122)	320	i.h.	NCT03410992
Papp KA 2018 (66)	IIb	PSO	Bimekizumab/IL-17A/F	250 (163/87)	64; 160; 320; 480	i.h.	NCT02905006
Glatt S 2018 (67)	Ib	PSA	Bimekizumab/IL-17A/F	53 (26/27)	40; 80; 160; 320; 560	i.v.	NCT02141763
Blauvelt A 2020 (68)	IIb	PSA	Bimekizumab/IL-17A/F	170 (N/A)	64; 160; 320	N/A	NCT03010527
Glatt S 2018 (69)	I	PSO	Bimekizumab/IL-17A/F	39 (30/9)	8; 40; 160; 480; 640	i.v.	NCT02529956
Svecova D 2019 (70)	I	PSO	M1095/IL-17A/F	41 (35/6)	30; 60; 120; 240	i.h.	NCT02156466

PSO, psoriasis; PSA, psoriatic arthritis; UC, ulcerative colitis; N/A, not available; M/F, Male/Female; i.v., intravenous injection; i.h., hypodermic injection.

### 3.2 Study quality

The SYRCLE risk of bias tool (**Supplementary Table 3**) was used to assess the risk of bias in each study; 90% of the research was of high quality. Four studies were included as high risk because their methods of data collection and results were potentially biased due to open labeling, unblinded researchers, or inconsistent patient administration, which may have affected the outcome assessment. The risk of bias was also assessed by a funnel plot constructed using the R language package. As shown in **Supplementary Figure 2**, the studies were concentrated and symmetrical, indicating that they had a small risk of bias. There was no evidence of publication bias according to Egger’s test (P=0.8927>0.05). A meta-regression analysis was used to re-examine the included studies without risk of bias (**Supplementary Figure 3**). A radial plot showed that the study is heterogeneous and therefore required subgroup analysis (**Supplementary Figure 4**). Sensitivity analyses showed that the overall incidence did not change significantly after removing any of the items individually; therefore, the model was considered stable (**Supplementary Figure 5**).

### 3.3 Outcomes

#### 3.3.1 Incidence of AEs with Anti-IL-17 BAs

The meta-analysis showed that 28,424 patients from 57 RCTs received anti-IL-17 drugs, and the incidence of AEs was 72.70% (95% CI 70.14–75.20., *I^2^* = 95%) (**Table 2**, **Supplementary Figure 6**). The incidence of AEs in patients with psoriasis (73.22% [95% CI 70.25–76.10, *I^2^* = 96%]) was comparable to that in patients with PSA (71.22% [95% CI 66.02–76.17, *I^2^* = 93%]) (**Table 2**, **Supplementary Figure 7**).

**Table 2:** Estimates of adverse events in the 57 included studies.

Trials	Any Grade Adverse Events					
	Studies	Patients of AEs	Total	Incidence/95% CI	*I^2^*	*P* Value
1. Diagnosis						
Psoriasis (PSO)	93	16,762	23,763	0.732 [0.702, 0.761]	96	<0.0001
Psoriasis arthritis (PSA)	33	3,291	4,661	0.712 [0.660, 0.762]	93	<0.01
2. Dose Adjustment						
< 80mg						
Secukinumab	1	84	99	0.848 [0.762, 0.913]	N/A	N/A
Brodalumab	2	48	77	0.626 [0.452, 0.785]	58	0.12
Bimekizumab	4	42	62	0.702 [0.567, 0.824]	44	0.15
80mg						
Ixekizumab	49	12674	18105	0.720 [0.680, 0.758]	97	<0.01
120-160mg						
Secukinumab	15	1793	2399	0.778 [0.701, 0.847]	94	<0.01
Brodalumab	4	338	613	0.565 [0.504, 0.625]	43	0.15
Bimekizumab	4	151	200	0.724 [0.523, 0.890]	88	<0.01
ABT-122	1	33	71	0.464 [0.345, 0.587]	N/A	N/A
210-300mg						
Brodalumab	7	425	641	0.733 [0.621, 0.832]	90	<0.01
ABT-122	1	31	73	0.425 [0.310, 0.546]	N/A	N/A
Secukinumab	19	3092	4102	0.770 [0.713, 0.822]	93	<0.01
> 300mg						
Bimekizumab	10	1011	1460	0.733 [0.645, 0.814]	90	<0.01
3. Courses of Medication						
12W	37	4,709	8,035	0.587 [0.576, 0.598]	43	<0.01
IL-17A	20	3,917	6,721	0.584 [0.572, 0.596]	48	<0.01
IL-17R	10	464	752	0.642 [0.581, 0.702]	55	0.02
IL-17A/F	7	328	562	0.585 [0.543, 0.626]	0	0.82
16W	6	851	1,460	0.586 [0.540, 0.631]	66	0.01
IL-17A	3	299	472	0.634 [0.590, 0.677]	0	0.6
IL-17R	2	339	639	0.531 [0.492, 0.569]	0	0.46
IL-17A/F	1	213	349	0.610 [0.557, 0.662]	N/A	N/A
20W	9	48	64	0.827 [0.621, 0.973]	59	0.01
IL-17A/F	9	48	64	0.827 [0.621, 0.973]	59	0.01
24W	13	1,404	2,086	0.707 [0.654, 0.757]	82	<0.01
IL-17A	12	1,313	1,982	0.689 [0.644, 0.733]	73	<0.01
IL-17R	1	91	104	0.875 [0.796, 0.932]	N/A	N/A
48W	7	1,342	2,381	0.592 [0.452, 0.725]	98	<0.01
IL-17A	7	1,342	2,381	0.592 [0.452, 0.725]	98	<0.01
52W	21	3,566	4,597	0.795 [0.756, 0.831]	86	<0.01
IL-17A	20	3,243	4,202	0.794 [0.752, 0.832]	86	<0.01
IL-17A/F	1	323	395	0.818 [0.776, 0.855]	N/A	N/A
>1Y	51	6,360	9,128	0.794 [0.745, 0.840]	97	<0.01
IL-17A	29	5,898	8,565	0.802 [0.746, 0.852]	97	<0.01
IL-17R	1	91	104	0.525 [0.361, 0.685]	N/A	N/A
IL-17A/F	5	371	459	0.801 [0.731, 0.864]	63	0.03
4. Frequency of Administration						
Q1wk						
ABT-122	2	64	144	0.444 [0.363, 0.527]	0	0.63
Q2wk						
lxekizumab	11	2139	3503	0.658 [0.590, 0.724]	91	<0.01
Secukinumab	1	357	387	0.922 [0.891, 0.947]	N/A	N/A
Brodalumab	13	909	1513	0.652 [0.578, 0.721]	85	<0.01
M1095	1	21	33	0.636 [0.451, 0.796]	N/A	N/A
Q4wk						
Secukinumab	36	4743	6433	0.758 [0.716, 0.798]	91	<0.01
lxekizumab	26	6798	10668	0.741 [0.698, 0.781]	96	<0.01
Brodalumab	1	30	41	0.731 [0.571, 0.858]	N/A	N/A
Bimekizumab	16	1131	1634	0.670 [0.627, 0.768]	87	<0.01
Q8wk						
Bimekizumab	1	77	100	0.770 [0.675, 0.848]	N/A	N/A
Q12wk						
lxekizuma	1	168	227	0.740 [0.678, 0.796]	N/A	N/A
5. Drugs						
IL-17A	88	17,841	25,051	0.735 [0.705, 0.764]	96	<0.0001
Ixekizumab	50	12,768	18,225	0.721 [0.682, 0.759]	97	<0.01
Secukinumab	36	5,009	6,682	0.768 [0.724, 0.809]	93	<0.01
ABT-122	2	64	144	0.444 [0.363, 0.527]	0	0.63
IL-17R	14	939	1,554	0.657 [0.588, 0.723]	84	<0.01
Brodalumab	14	939	1,554	0.657 [0.588, 0.723]	84	<0.01
IL-17A/F	24	1,273	1,819	0.731 [0.667, 0.791]	85	<0.01
Bimekizumab	23	1,252	1,786	0.736 [0.669, 0.799]	85	<0.01
M1095	1	21	33	0.636 [0.451, 0.796]	N/A	N/A
6. Types of AEs						
Type-α	106	8,363	25,577	0.334 [0.285, 0.385]	98	<0.0001
Type-β	108	6,113	25,568	0.223 [0.191, 0.257]	96	<0.0001
Type-γ	69	733	19,753	0.047 [0.036, 0.059]	93	<0.01
Type-δ	35	153	14,744	0.010 [0.007, 0.013]	66	<0.01
Type-ϵ	32	117	7,092	0.016 [0.010, 0.023]	69	<0.01
6.1 Type α						
Viral URI	8	325	1,876	0.155 [0.088, 0.235]	95	<0.01
URI	82	1,828	22,861	0.078 [0.066, 0.092]	89	<0.01
Diarrhea	32	336	7,189	0.040 [0.030, 0.052]	82	<0.01
Headache	69	1,397	19,722	0.060 [0.052, 0.068]	77	<0.01
Back pain	25	331	6,897	0.042 [0.034, 0.052]	66	<0.01
Urinary tract infection	24	132	3,895	0.033 [0.022, 0.045]	73	<0.01
Candida infections	46	533	14,253	0.035 [0.026, 0.046]	86	<0.01
Cough	19	184	4,028	0.041 [0.030, 0.054]	59	<0.01
Arthralgia	41	753	15,268	0.046 [0.037, 0.056]	79	<0.01
Nausea	17	97	3,034	0.033 [0.020, 0.049]	65	<0.01
Oropharyngeal pain	13	156	3,462	0.032 [0.020, 0.045]	62	<0.01
Infections	55	5,008	18,842	0.332 [0.276, 0.390]	99	<0.0001
Serious infections	26	91	7,029	0.011 [0.008, 0.015]	27	0.1
Fatigue	4	26	538	0.045 [0.028, 0.065]	0	0.56
Influenza	3	68	1,145	0.051 [0.029, 0.078]	50	0.14
Pain in extremity	5	20	257	0.074 [0.043, 0.111]	0	0.49
Dizziness	3	3	72	0.022 [0.000, 0.085]	9	0.33
Vomiting	4	20	426	0.046 [0.027, 0.069]	0	0.98
Gastrointestinal disorders	5	12	92	0.105 [0.039, 0.189]	0	0.52
Abdominal pain	2	3	12	0.246 [0.027, 0.550]	0	0.56
6.2 Type β						
Injection-site reaction	59	1,866	19,736	0.083 [0.063, 0.105]	95	<0.01
Bronchitis	25	333	5,964	0.051 [0.041, 0.063]	68	<0.01
Injection-site erythema	10	125	2,773	0.053 [0.032, 0.078]	83	<0.01
Allergic reaction/hypersensitivity events	24	228	5,253	0.042 [0.030, 0.057]	83	<0.01
Nasopharyngitis	97	3,715	23,662	0.137 [0.118, 0.159]	92	<0.01
Sinusitis	15	157	3,390	0.046 [0.035, 0.058]	48	0.02
Pruritus	19	209	5,102	0.061 [0.033, 0.095]	83	<0.01
Decreased neutrophils	10	94	2,378	0.025 [0.008, 0.049]	92	<0.01
Eczema	9	67	1,230	0.080 [0.042, 0.126]	69	<0.01
Urticaria	6	20	232	0.082 [0.048, 0.124]	0	0.66
Neutropenia	9	17	998	0.019 [0.000, 0.066]	68	<0.01
6.3 Type γ						
Hepatic event	27	219	6,668	0.034 [0.025, 0.046]	79	<0.01
Hypertension	31	308	6,689	0.040 [0.033, 0.048]	51	<0.01
Cerebro‐cardiovascular events	36	225	14,588	0.015 [0.011, 0.020]	80	<0.01
Cytopenia	4	5	239	0.017 [0.002, 0.041]	0	0.49
PSA/PSO	12	42	1,207	0.021 [0.011, 0.032]	26	0.19
Crohn’s disease	13	20	9,570	0.002 [0.001, 0.004]	14	0.3
Ulcerative colitis	4	5	1,329	0.004 [0.001, 0.008]	0	0.94
Inflammatory bowel disease	14	21	8,807	0.002 [0.001, 0.003]	0	0.94
6.4 Type Σ						
Depression	22	73	4,930	0.014 [0.010, 0.018]	26	0.13
Nervous system disorders	5	19	92	0.188 [0.104, 0.285]	0	0.81
7. Severity of AE						
TEAE	44	7485	11532	0.713 [0.662, 0.761]	98	<0.0001
SAE	1516	24749	24794	0.045 [0.031, 0.060]	91	<0.01

AEs, adverse events; URI, Upper Respiratory Tract Infection; PSA, psoriatic arthritis; PSO, psoriasis; TEAE, Treatment-emergent adverse events; SAE, Serious adverse events.

#### 3.3.2 Effect of doses of anti-IL-17 BAs

A subgroup analysis showed that doses less than 80 mg resulted in the lowest incidence of AEs for brodalumab at 62.6% (95% CI 0.452– 0.785, *I^2^* = 58%), and doses ranging from 120–160 mg were associated with the lowest incidence of AEs for ABT-122 at 46.4% (95% CI 0.345–0.587) and the highest incidence of AEs for secukinumab at 77.8% (95% CI 0.701–0.847, *I^2^* = 94%). At doses ranging from 210–300 mg, the lowest incidence of AEs due to ABT-122 was 42.5% (95% CI 0.310–0.546) (**Table 2**, **Supplementary Figure 8, 8.1–8.7**).

#### 3.3.3 Effect of duration of anti-IL-17 treatment

**Figure 2:**
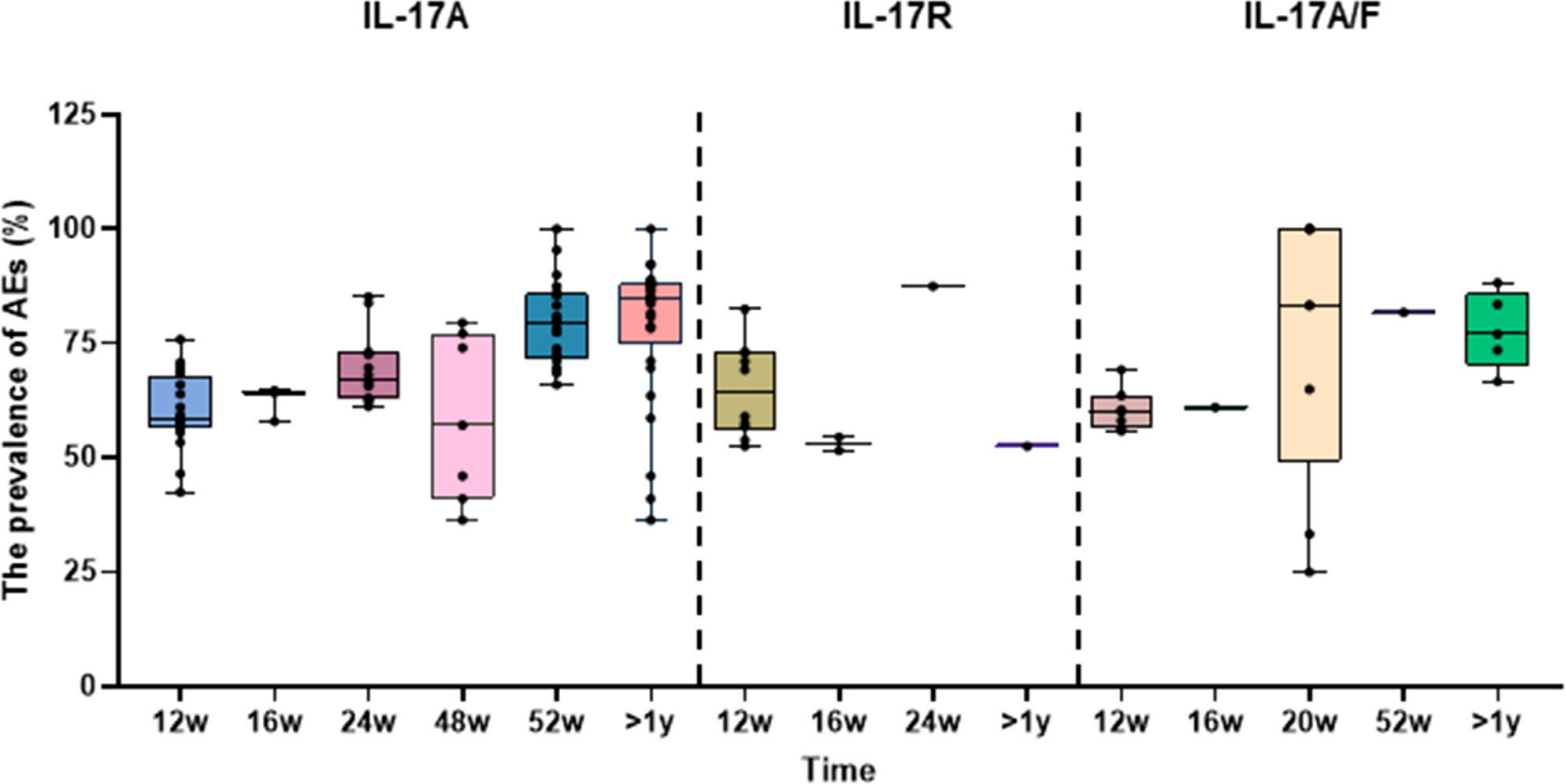
Box diagram of subgroup analysis. Box diagram of the incidence of changes in adverse events among different medication courses.

A subgroup analysis of the treatment duration in 57 studies showed that anti-IL-17A and anti-IL-17A/F BAs were relatively safe when administered for less than 48 weeks, and the incidence of AEs was approximately 58–69%. However, the incidence of AEs reached 80% for treatment durations longer than 52 weeks. The 20-week data were not included because the number of patients was inadequate. In addition, due to the small sample size in the clinical trials, the AEs of anti-IL-17R BAs were insufficient to determine the effect of treatment duration (**Table 2**, **Supplementary Figure 9**, and **Supplementary Figure 9.1–9.7**). The boxplot clearly and intuitively reflects the effect of treatment duration on the safety of each anti-IL-17 BA (**Figure 2**).

#### 3.3.4 Effect of dosing interval of anti-IL-17 BAs

A subgroup analysis of dosing intervals showed that Secukinumab had a 92.2% (95% CI 0.891–0.947) incidence of AEs when administered at a mean interval of 2 weeks, and the incidence of AEs decreased to 75.8% (95% 0.716–0.798, *I^2^* = 91%) when the interval was prolonged to 4 weeks. The incidence of AEs did not differ significantly when the dosing intervals of other BAs were changed within the safe limits (**Table 2**, **Supplementary Figure 10**, **10.1-10.5**).

#### 3.3.5 Targeted subunits and commercial drugs

The subgroup analyses showed that the incidence of AEs associated with anti-IL-17A and anti-IL-17A/F BAs was similar, both of which were higher than those associated with anti-IL-17R BAs. Further subgroup analysis showed that the incidence of AEs was 73.48% (95% CI 70.48–76.38, *I^2^* = 96%) for the anti-IL-17A BAs and 73.12% (95% CI 66.71–79.14, *I^2^* = 83%) for the anti-IL-17A/F BAs, which were not significantly different. These were both higher than the incidence of AEs for BAs against IL-17R, which was 65.66% (95% CI 58.75–72.26, *I^2^* = 84%) (**Table 2**, **Supplementary Figure 11**). Further subgroup analyses were performed to assess BAs with different targets, for which the incidence of AEs were: 72.10% (95% CI 68.18–75.88, *I^2^* = 97%) for ixekizumab, 76.79% (95% CI 72.44–80.88, *I^2^* = 93%) for secukinumab, 44.44% (95% CI 36.35–52.68, *I^2^* = 0%) for ABT-122, 65.66% (95% CI 58.75–72.26, *I^2^* = 84%) for brodalumab, 73.61% (95% CI 69.92–79.87, *I^2^* = 84%) for bimekizumab, and 63.64% (95% CI 45.13–79.60) for M1095 (**Table 2**, **Supplementary Figure 12**).

#### 3.3.6 Incidence of the five types of AEs

A subgroup analysis of five types of AEs showed that type α was the most common (33.4%), followed by types β (22.3%), γ (4.7%), δ(1.0%), and ϵ (1.6%). Detailed values are listed in **Table 2** and **Supplementary Figure 13**.

##### 3.3.6.1 Type α AEs

**Figure 3:**
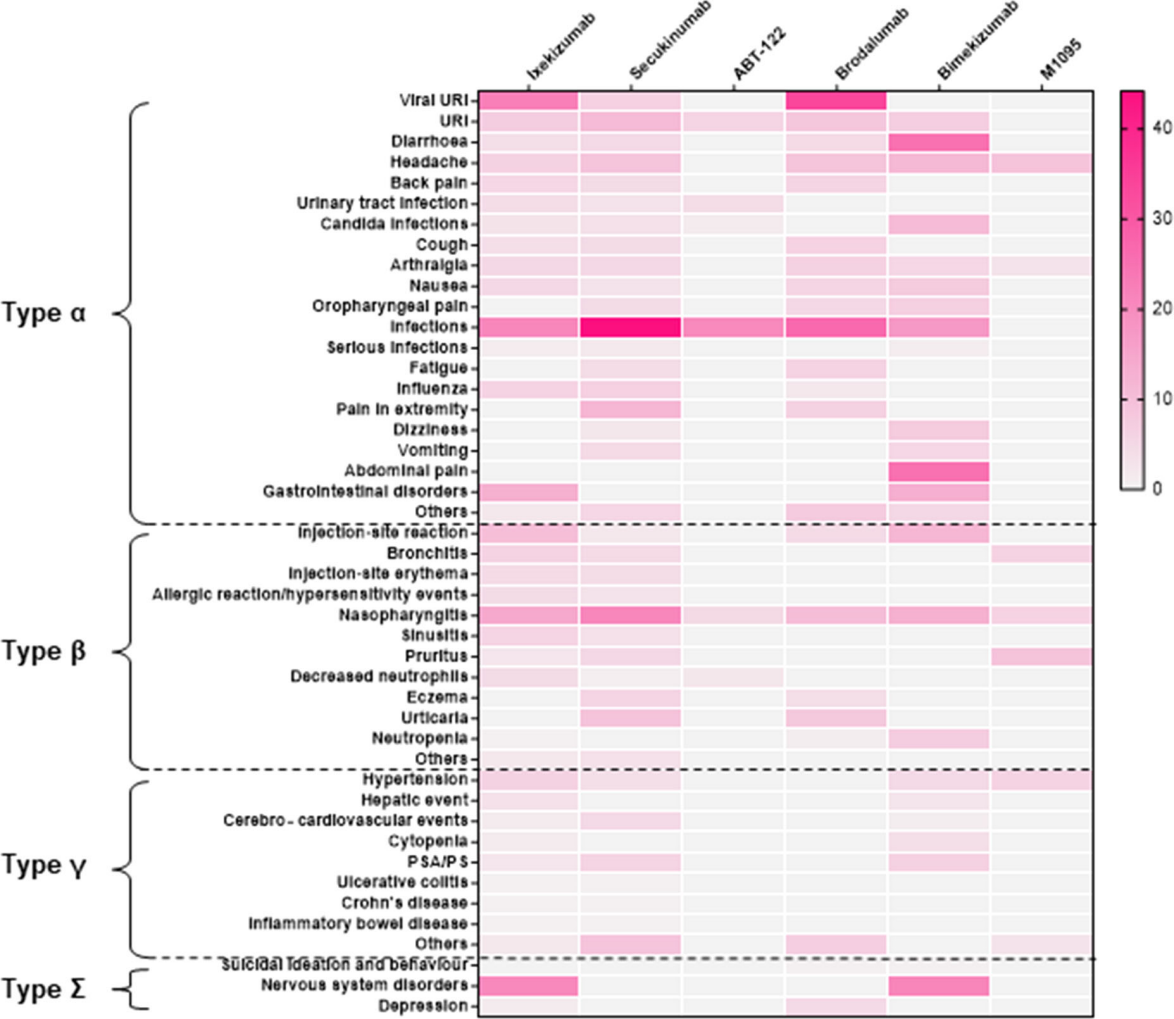
Heat map of different types of symptoms caused by different drugs. Red areas indicate greater relative probability of occurrence and lighter-colored areas indicate a slight or null relative probability of occurrence.

According to the subgroup analyses, anti-IL-17 BAs were the most likely to cause type α AEs and had the highest probability of causing infection, with an incidence rate of 26.98%. Biologics against IL-17A/F most frequently induced diarrhea and abdominal pain, both occurring in 25% of the patients. The most frequent AE induced by IL-17R BAs was viral upper respiratory tract infection, with an incidence as high as 33.65%, followed by infection (26.76%). Detailed values are listed in **Table 2**, **Supplementary Table 4** and **Supplementary Figure 14**. The dark areas of the heat map shown in **Figure 3** represent a high incidence of AEs and vice versa.

##### 3.3.6.2 Type β AEs

The incidence of type β AEs was second only to that of type α. Biologics targeting IL-17A, IL-17A/F, and IL-17R were the most common causes of nasopharyngitis, with incidence rates of 16.05%, 12.62%, and 10.27%, respectively. The second most common type β AEs caused by anti-IL-17A and anti-IL-17A/F BAs were injection site reactions with an incidence of 9.52% and 11.54%. Anti-IL-17R was likely to induce urticaria, with an incidence of 8.08%. Patients treated with anti-IL-17A/F BAs were also prone to pruritus, with an incidence of 9.09%. The incidence of other type β AEs, including sinusitis and neutropenia, was less than 3–6% (**Table 2**, **Supplementary Table 4**, **Supplementary Figure 14**).

##### 3.3.6.3 Type γ AEs

Anti-IL-17A and anti-IL-17A/F BAs were more likely to induce type γ AEs than anti-IL-17R BAs. Anti-IL-17A BAs were most likely to induce hypertension (4.59%) and hepatic events (3.41%), and anti-IL-17A/F BAs were most likely to induce psoriasis and PSA (6.11%), followed by hypertension (4.82%) (**Table 2**, **Supplementary Table 4**, **Supplementary Figure 14**).

##### 3.3.6.4 Type ϵ AEs

Anti-IL-17A and anti-IL-17A/F BAs were also more likely to induce type ϵ adverse reactions than anti-IL-17R BAs. The most common were neurological diseases, with these two different biologics causing these AEs with incidence rates of 20.37% and 21.05% (**Table 2**, **Supplementary Table 4**, **Supplementary Figure 14**).

#### 3.3.7 Severity of AE

According to their severity, AEs are classified as treatment-emergent AEs (TEAEs) or serious AEs (SAEs). An SAE is frequently described as an adverse reaction that necessitates the discontinuation of therapy. This study revealed that when anti-IL-17 BAs were used to treat moderate to severe psoriasis, the incidence of TEAEs was 71.32%, whereas that of SAEs was 4.46% (**Supplementary Figures 15**, **16**).

## 4 Discussion

Psoriasis is one of the most common chronic inflammatory skin diseases worldwide. Repeated disease recurrence is a major bottleneck in current treatments. A survey study showed that the top treatment needs of patients with psoriasis in the Asia-Pacific region were long-term remission and reduced recurrence. In this region, 68% of patients with psoriasis believe that the rapid clearance of skin lesions is the key to psoriasis treatment options (71). Similarly, the 2018 edition of the Chinese Psoriasis Diagnosis and Treatment Guidelines considers the prevention of recurrence as an important goal of psoriasis treatment and emphasizes that reducing disease progression and recurrence and improving quality of life are crucial for patients (72). However, traditional methods of treating psoriasis can no longer meet the needs of patients with moderate-to-severe psoriasis. The public has become aware of BAs due to their efficacy, but the safety of these treatments is a problem that cannot be ignored. The main adverse reactions to almost all anti-IL-17 BAs are infection, diarrhea, headache, and back pain. Brodalumab, which was co-developed by AstraZeneca and Amgen, caused occasional suicidal ideation or behavioral events in patients during 2015, but a recent study showed no link between brodalumab and suicide (73). However, a meta-analysis of 163 RCTs and 46 extension studies showed that biologics are associated with significantly higher rates of adverse events Compared to the control group (74). In addition, psoriasis often presents with comorbidities such as cardiovascular disease, abnormal lipid metabolism, chronic obstructive pulmonary disease, and inflammatory bowel disease. Therefore, the safety of BAs must be considered not only for psoriasis but also with regard to its comorbidities.

To our knowledge, this is the first systematic review and meta-analysis to comprehensively evaluate AEs caused by IL-17 BAs in the treatment of psoriasis. We included all relevant RCTs to date, which lends credibility to the data. **Table 2** lists all the AE-related information retrieved from the 57 studies included in the analysis. Our study found a higher incidence of AEs associated with anti-IL-17A (73.48%) and anti-IL-17A/F (73.12%) than with anti-IL-17R (65.66%). With treatment durations longer than one year, the incidence of adverse reactions was as high as 79%, and long-term use of medication had the potential to lead to mental disorders. The most common AEs caused by BAs were type α, with an incidence of 33.52%, of which infection was the most common. This was followed by type β adverse reactions with an incidence rate of 22.29%, of which nasopharyngitis was the most common. The incidence rates of type γ, δ, and **ϵ** AEs were 4.72%, 1.01%, and 1.66%, respectively. Hepatic events were the most common among type γ AEs, which occurred in 3.44% of patients. However, a longitudinal cohort study of 1061 patients with PSA showed liver abnormalities in these patients; therefore, biologics are not the sole contributors to liver events (75). Depression was the most common type **ϵ** adverse reaction induced by BAs, with an incidence of 1.37%. This may be explained by studies that have shown that mental disorders are comorbid with psoriasis, inflammation and depression share certain molecular mechanisms, and inflammatory factors such as IL-6 are significantly increased in patients with depression (1). However, long-term medication use may also lead to mental disorders. Therefore, regardless of whether BAs are used, doctors should pay more attention to the psychological state of patients and provide psychological treatment when necessary to prevent the deterioration of the condition. The lowest incidence of AEs was type δ (1.01%), which includes adverse reactions such as malignant tumors and acne. Two studies (76, 77) systematically reviewed biologics in the treatment of rheumatoid arthritis and malignancies, and evidence suggests that biologics not only do not cause malignancy but may also reduce the incidence of tumors due to their inhibitory effect on inflammation. However, there is currently no relevant evidence in patients with psoriasis, and we believe that BAs are safe and effective and that the occurrence of malignant tumors is likely unrelated to BAs. Our study found that only 4.46% of patients treated with anti-IL-17 BAs experienced SAEs, at which point the treatment had to be discontinued and the rest of the adverse events could be managed during the remission phase.

This systematic review and meta-analysis evaluated the safety of IL-17-related biologics in the treatment of psoriasis and PSA by summarizing all relevant RCTs to date to facilitate better clinical decision-making. The quality of the included studies was high. Additionally, this study included more than 28,000 patients. The large sample size and use of high-quality studies suggest that our data analysis is convincing and the evidence sufficient and reliable. Each author independently screened the literature and extracted article data, which ensured that it was reliable and reduced the occurrence of errors. Finally, we analyzed and compared multiple subgroups with adverse reactions and fully mined the data. However, this study has some limitations. First, the included studies did not exclude the underlying diseases of the patients and could not fully determine whether some AEs were caused by BAs. Second, individual articles did not clearly define SAEs, which are divided into severe and serious AEs, which may have produced slight errors in the data analysis. Third, some articles were unclear about the definition of certain AEs, such as skin system diseases and musculoskeletal diseases. To ensure accuracy, we discarded these data. Fourth, there are too few IL-17R-related studies, and the evidence is not sufficient. Therefore, it is recommended to conduct large-scale, multi-center clinical trials to confirm the safety of anti-IL-17R BAs in the future. Biologics will likely become the focus of psoriasis treatment in the future; however, shortcomings remain. In the future, it is necessary to further clarify the mechanism of common adverse reactions caused by related monoclonal antibodies and their impact on the comorbidities of psoriasis to reduce their occurrence and benefit the majority of patients.

### 4.1 Conclusion

Among the adverse events caused by the use of anti-IL-17 agents, cytokine release syndrome and allergic reactions were the most common. Given that immunologic agents are increasingly used in the clinical setting, their AEs should receive more attention.

## Data availability statement

The original contributions presented in the study are included in the article/**Supplementary Material**. Further inquiries can be directed to the corresponding authors.

## Author contributions

XL and BL proposed and designed the study; XL obtained funding; JW and LL retrieved and selected the data; JW, LL, and CW extracted the data; SH and YR assessed the quality of all studies; NL, MZ, and XS performed the statistical analyses of all the data; JW and CW drafted the manuscript; and XL revised the manuscript. All authors contributed to the article and approved the submitted version.
