# Supplementary material for: Adverse events associated with anti-IL-17 agents for psoriasis and psoriatic arthritis: a systematic scoping review
Source: Front Immunol. 2023 Jan 31;14:993057. doi: 10.3389/fimmu.2023.993057 (PMC9928578; doi:10.3389/fimmu.2023.993057)

**Supplementary Figure 1.** PRISMA 2009 flow diagram.


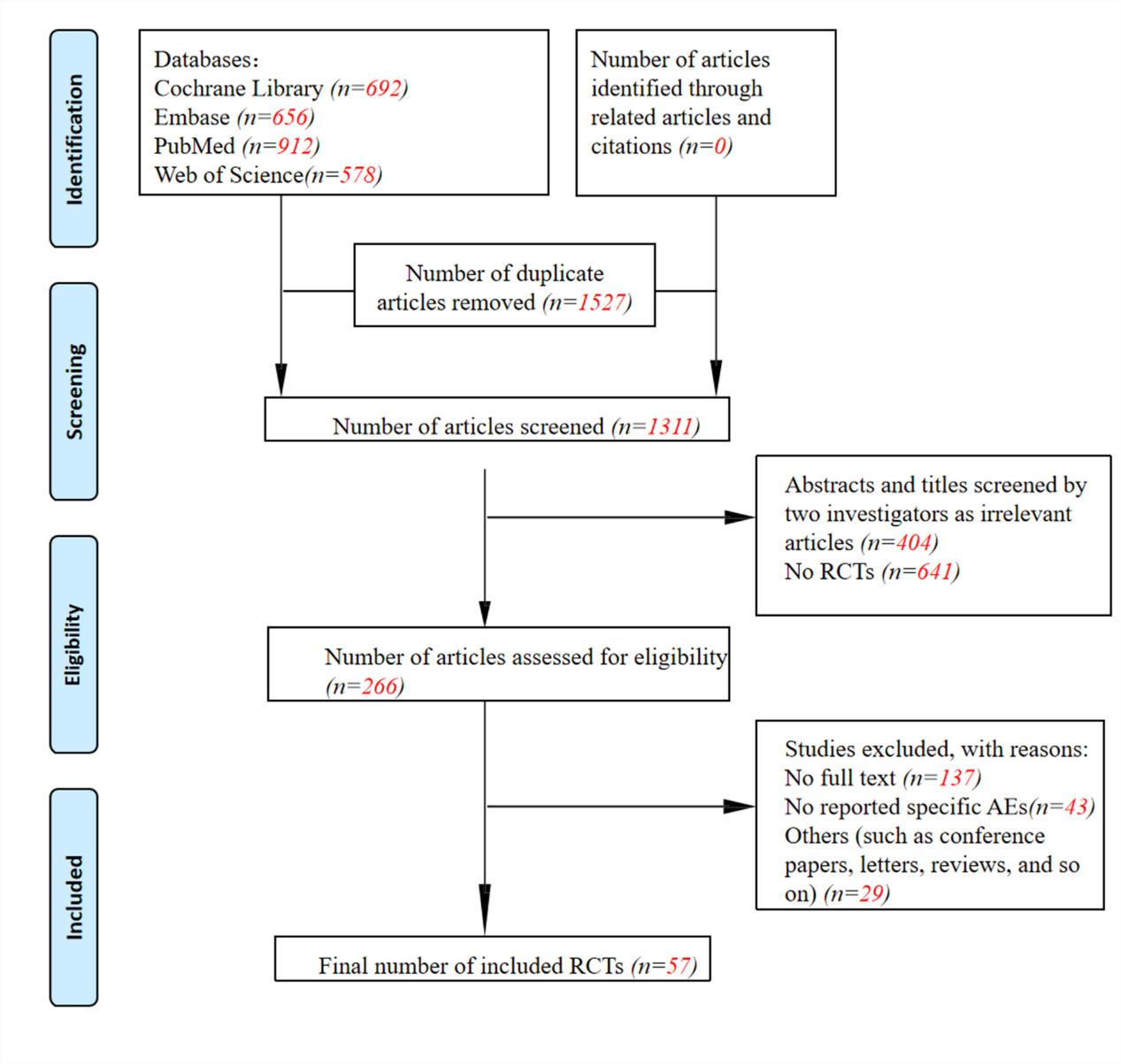


**Supplementary Figure 2.** Funnel plot.


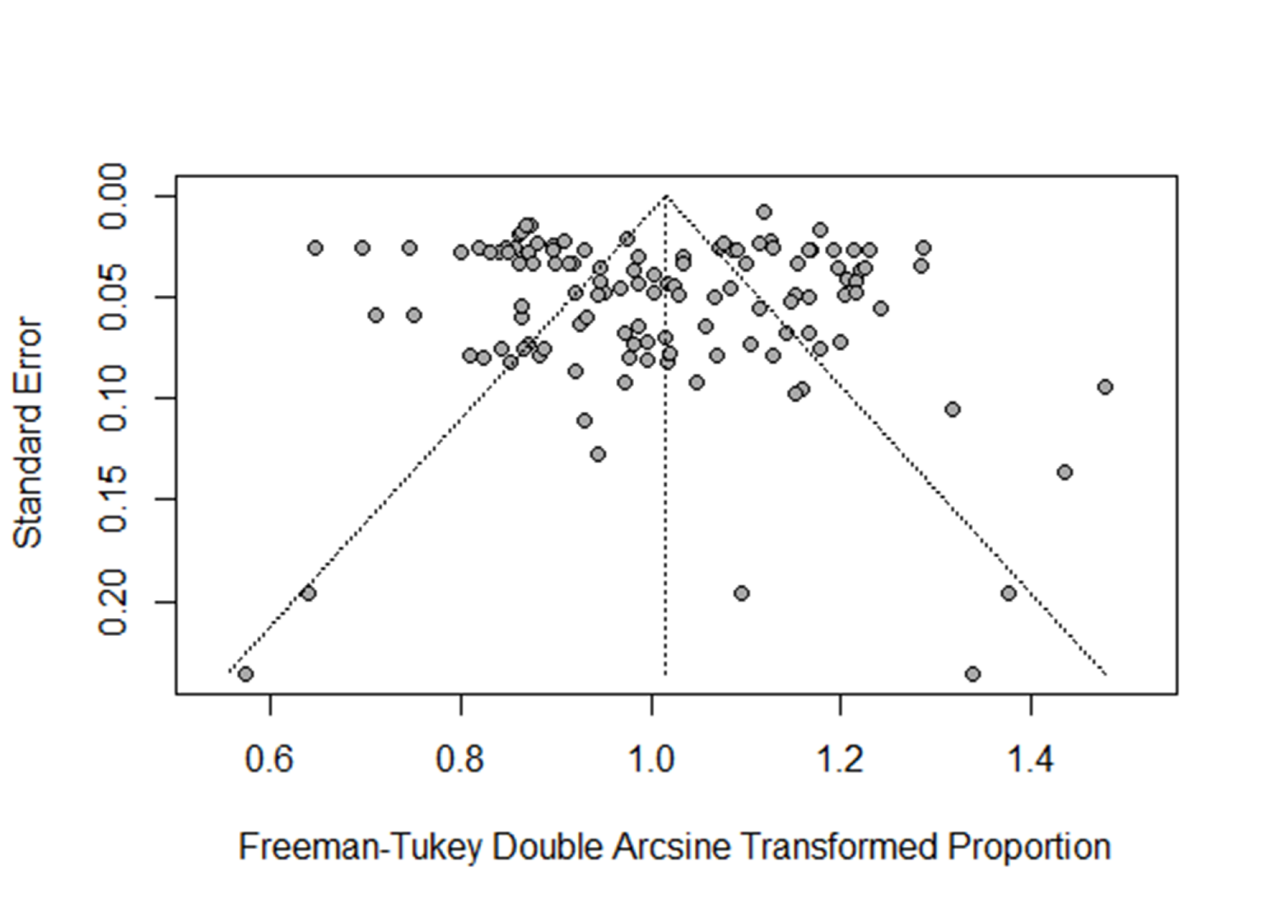


**Supplementary Figure 3.** Egger’s test.


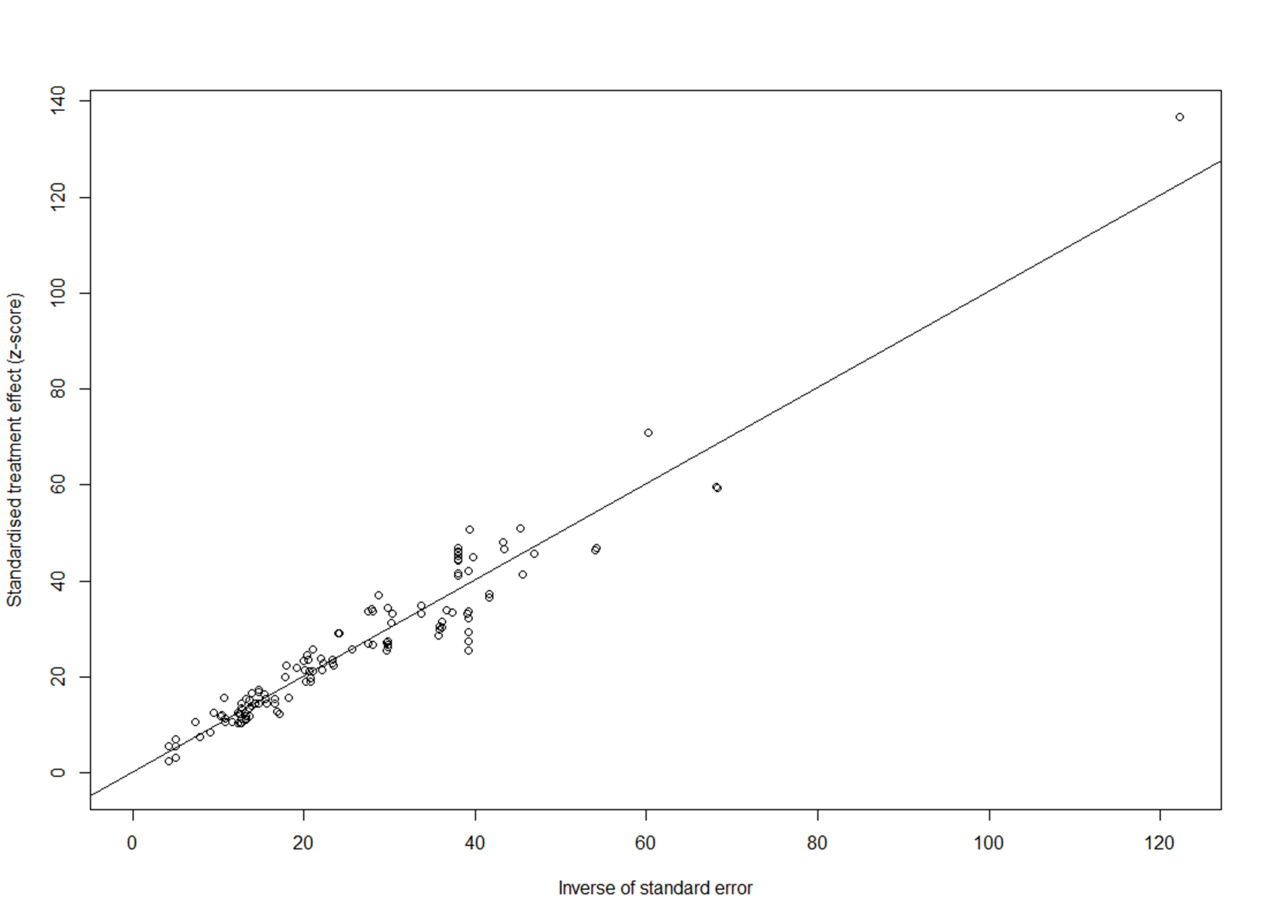


**Supplementary Figure 4.** Radial plot.


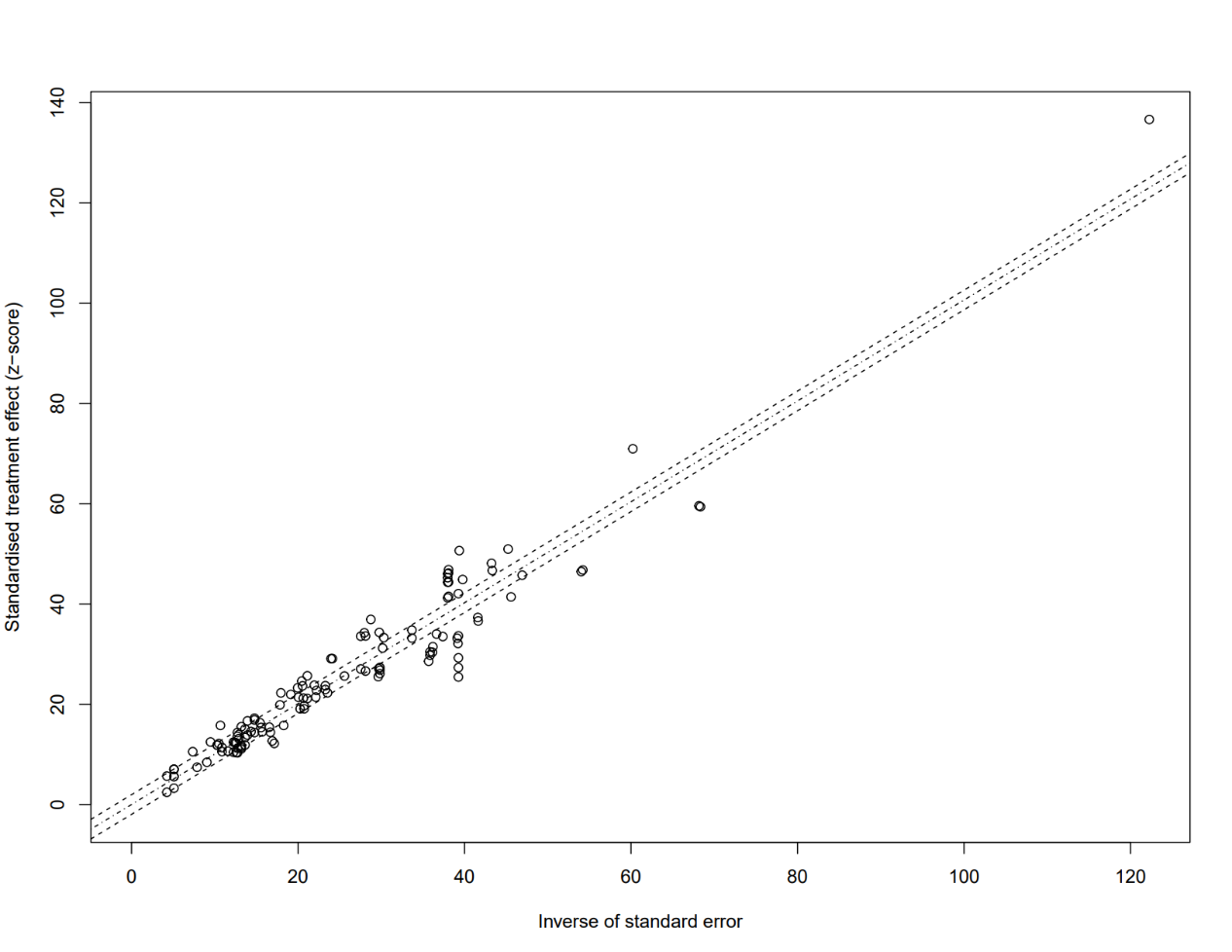


**Supplementary Figure 5.** Sensitivity analysis.


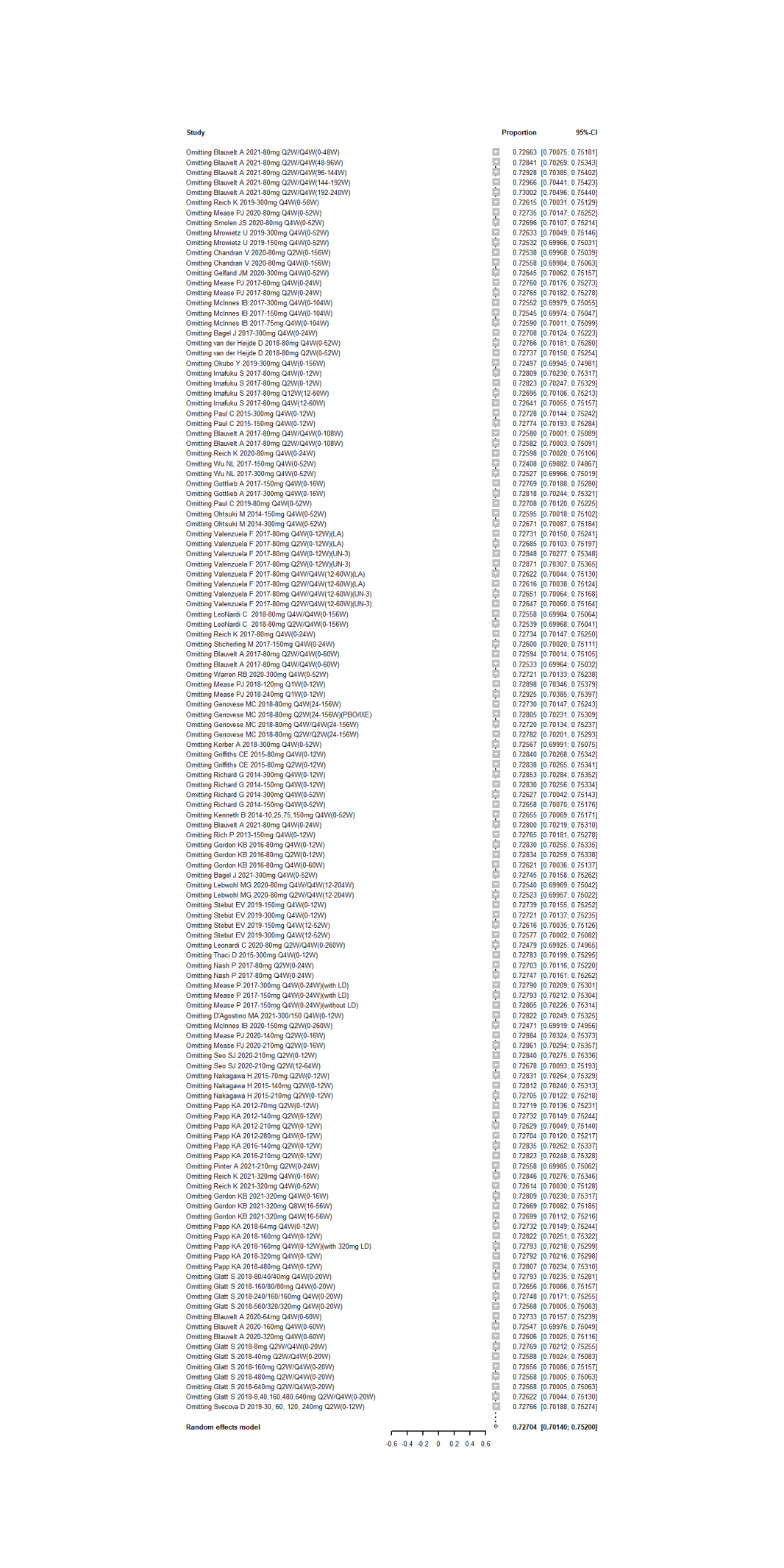


**Supplementary Figure 6.** Incidence of all adverse events caused by anti-IL-17 agents in all patients.


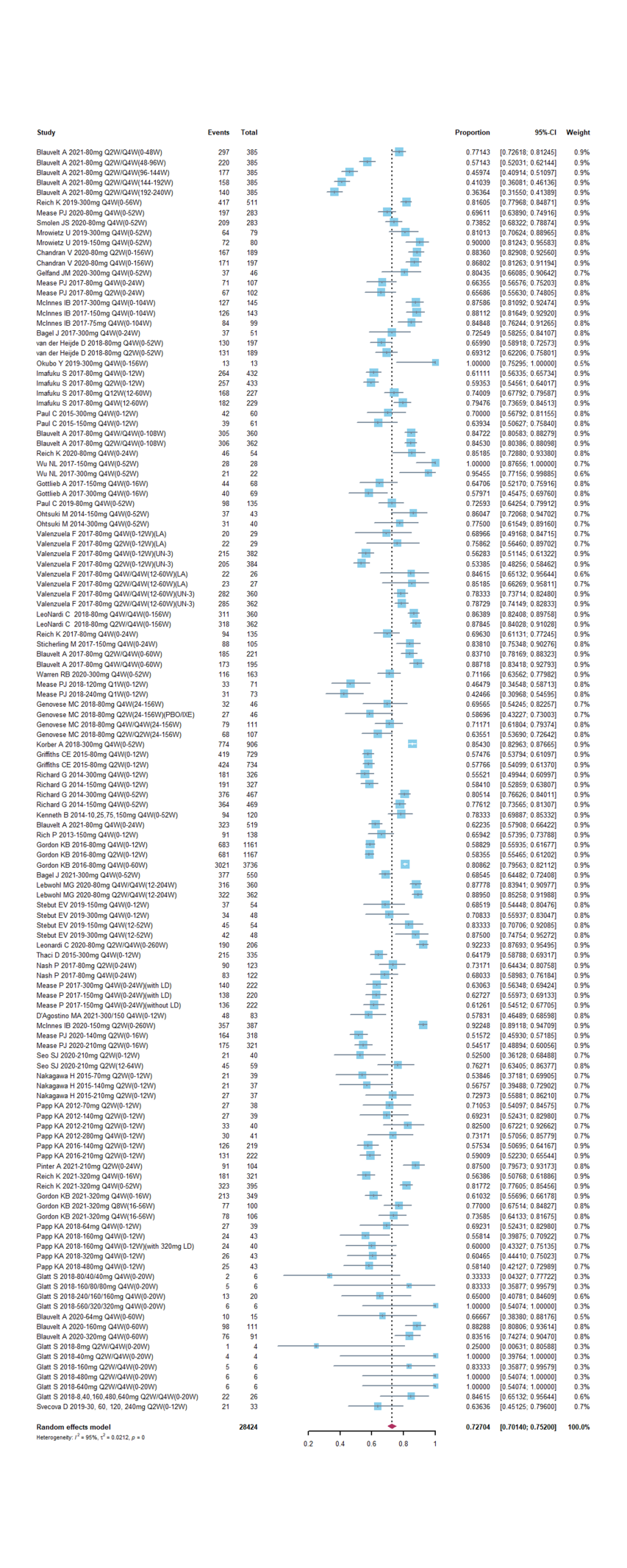


**Supplementary Figure 7.** Incidence of adverse events caused by anti-IL-17 agents in different diseases.


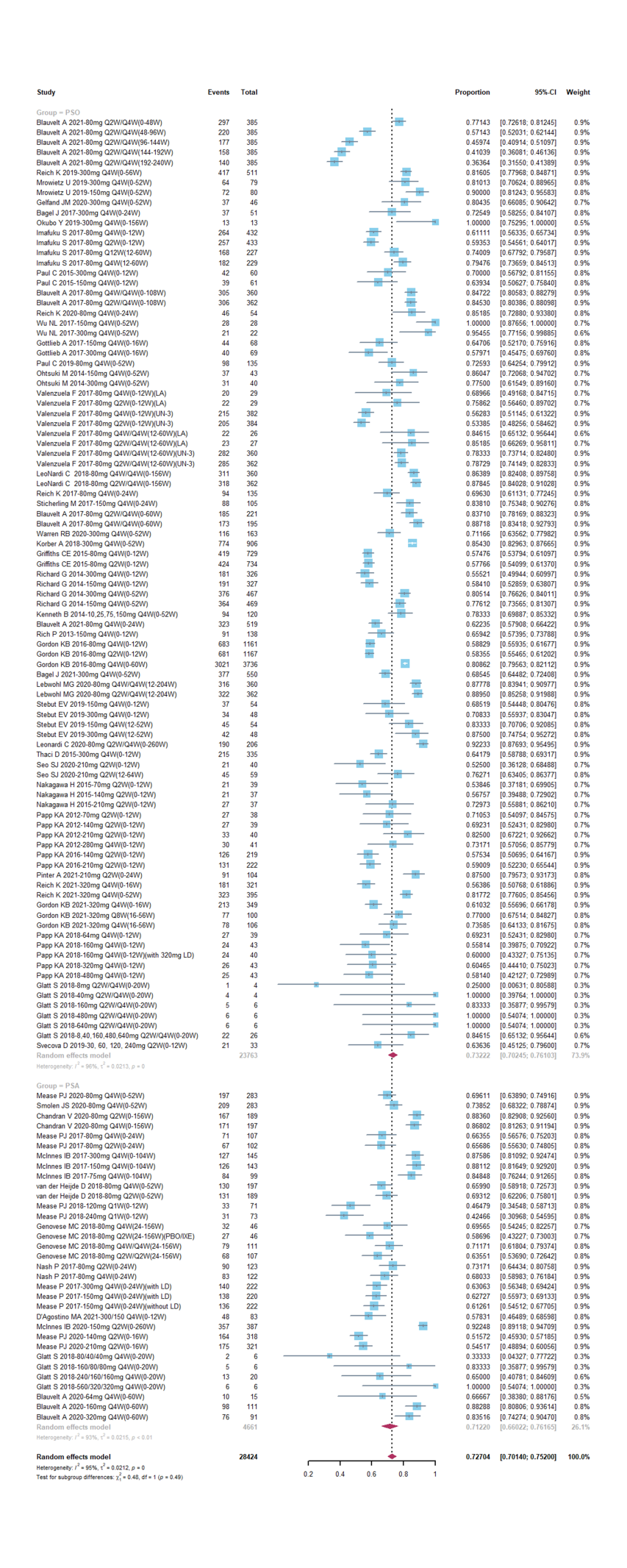


**Supplementary Figure 8.** Incidence of adverse events caused by anti-IL-17 agents at different doses.


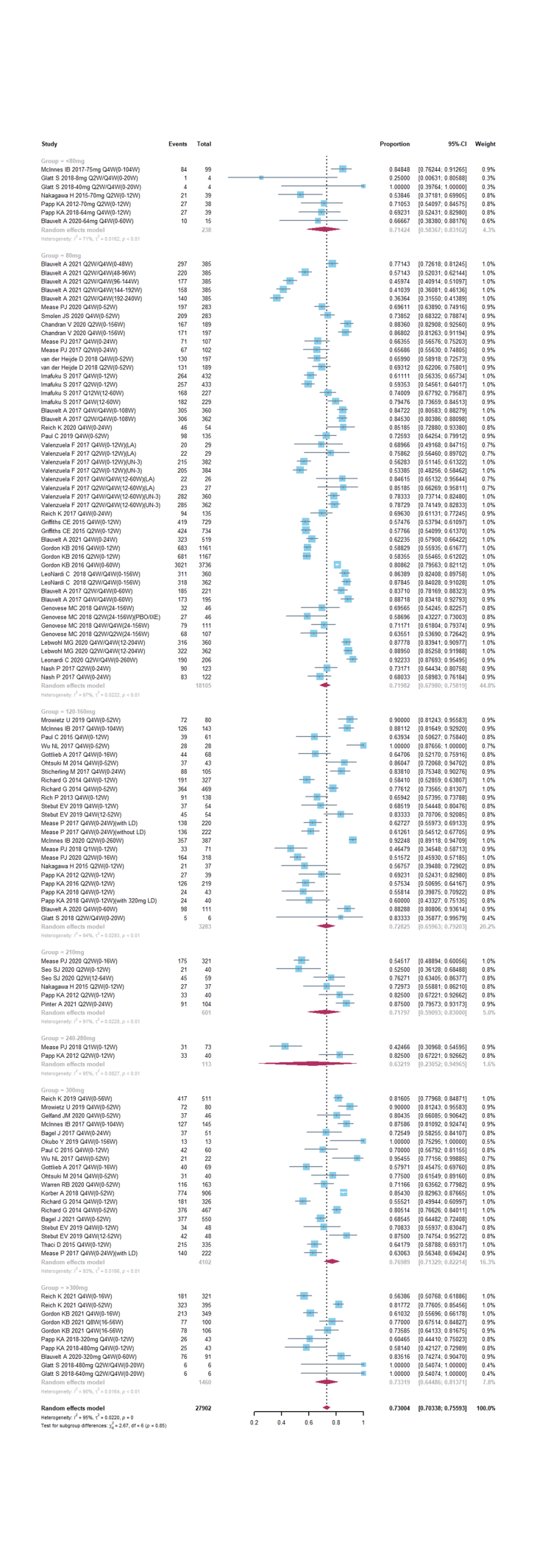


**Supplementary Figure 8.1–8.7** Subgroup analysis of adverse events caused by anti-IL-17 drugs at different doses.


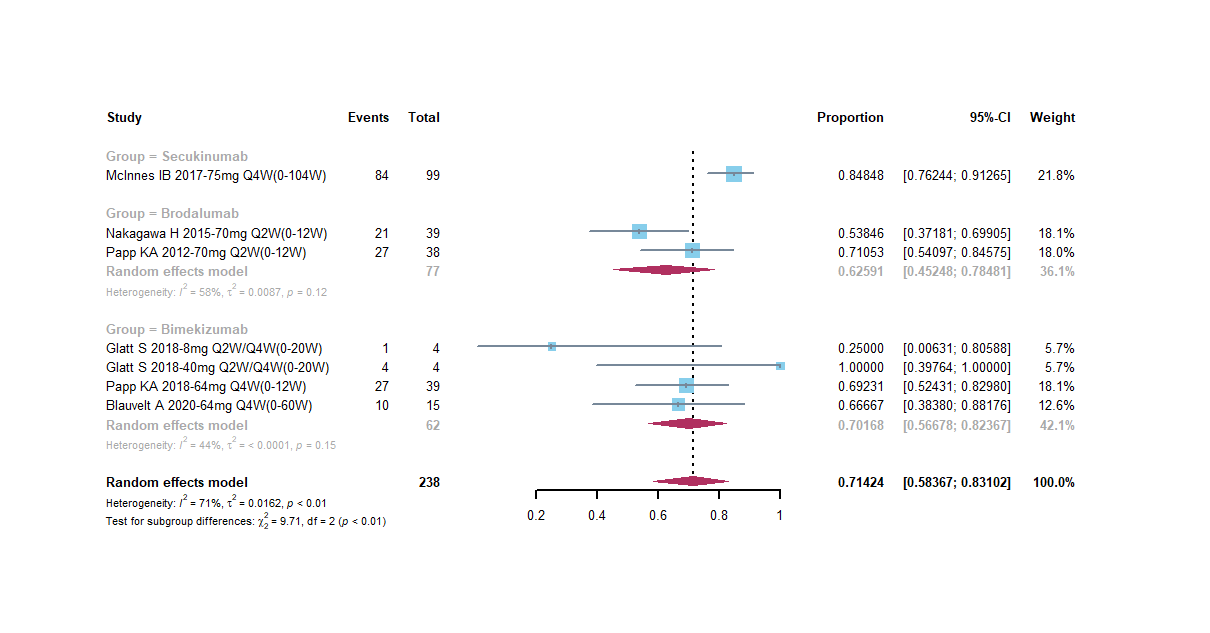

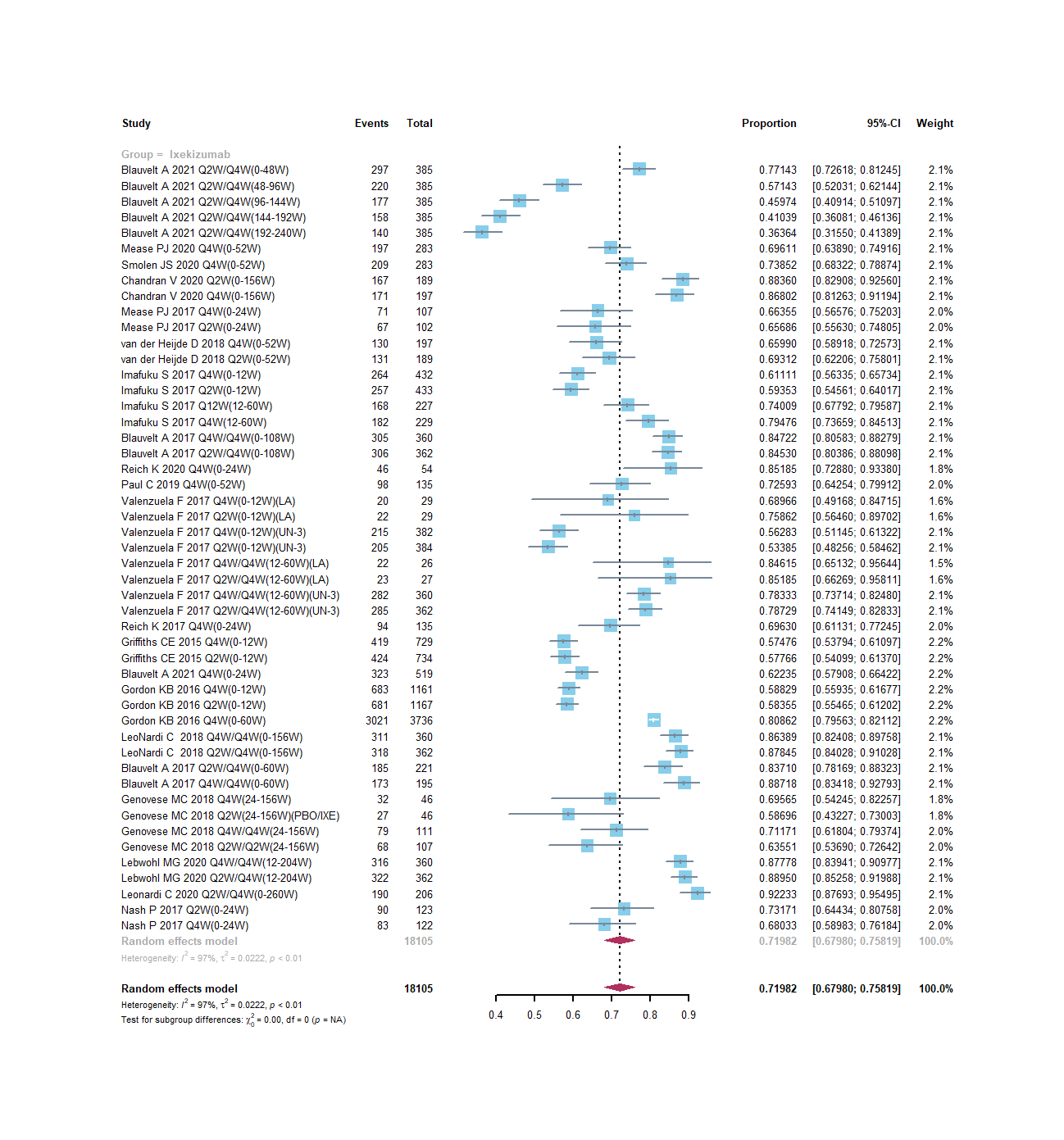

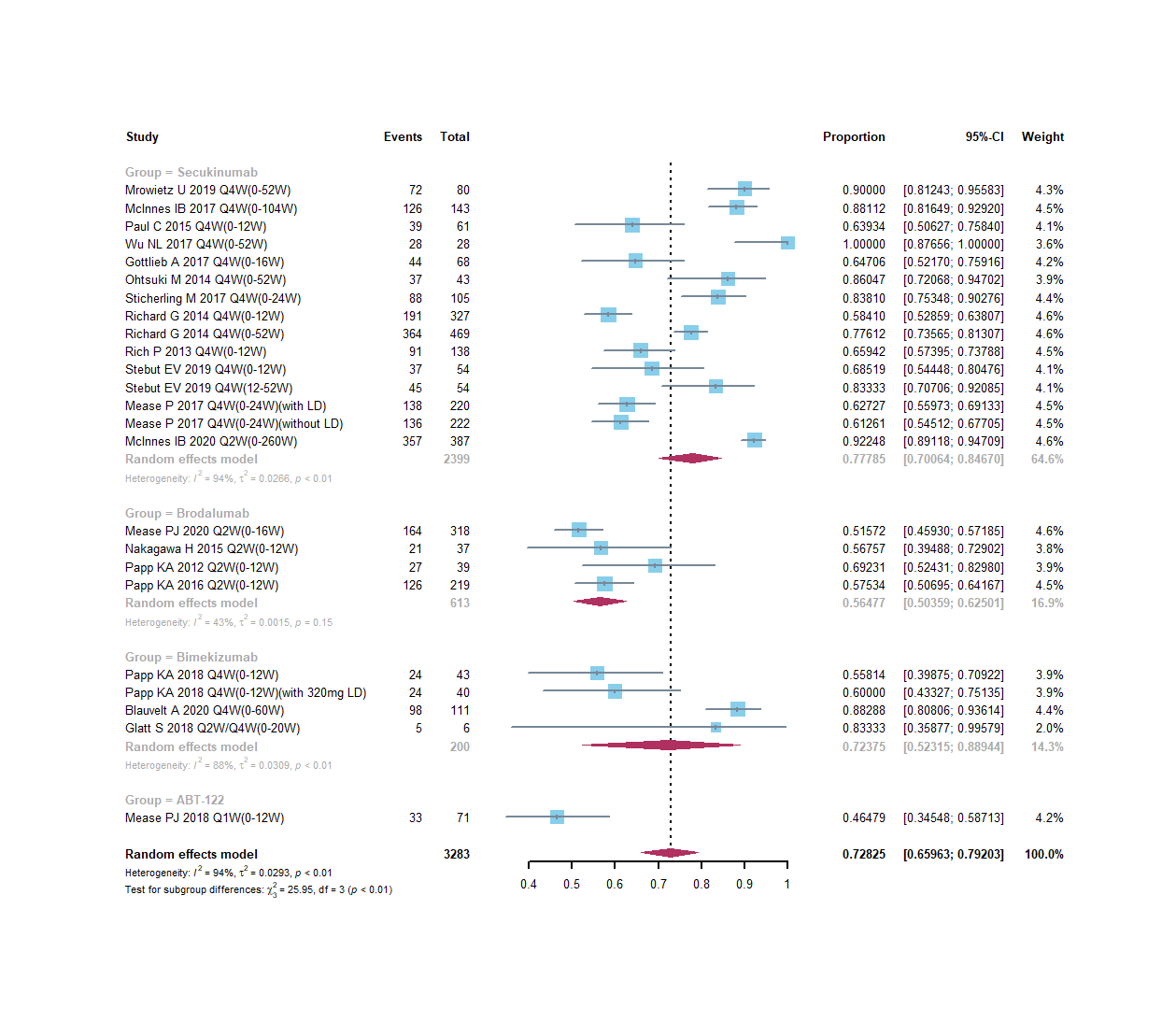

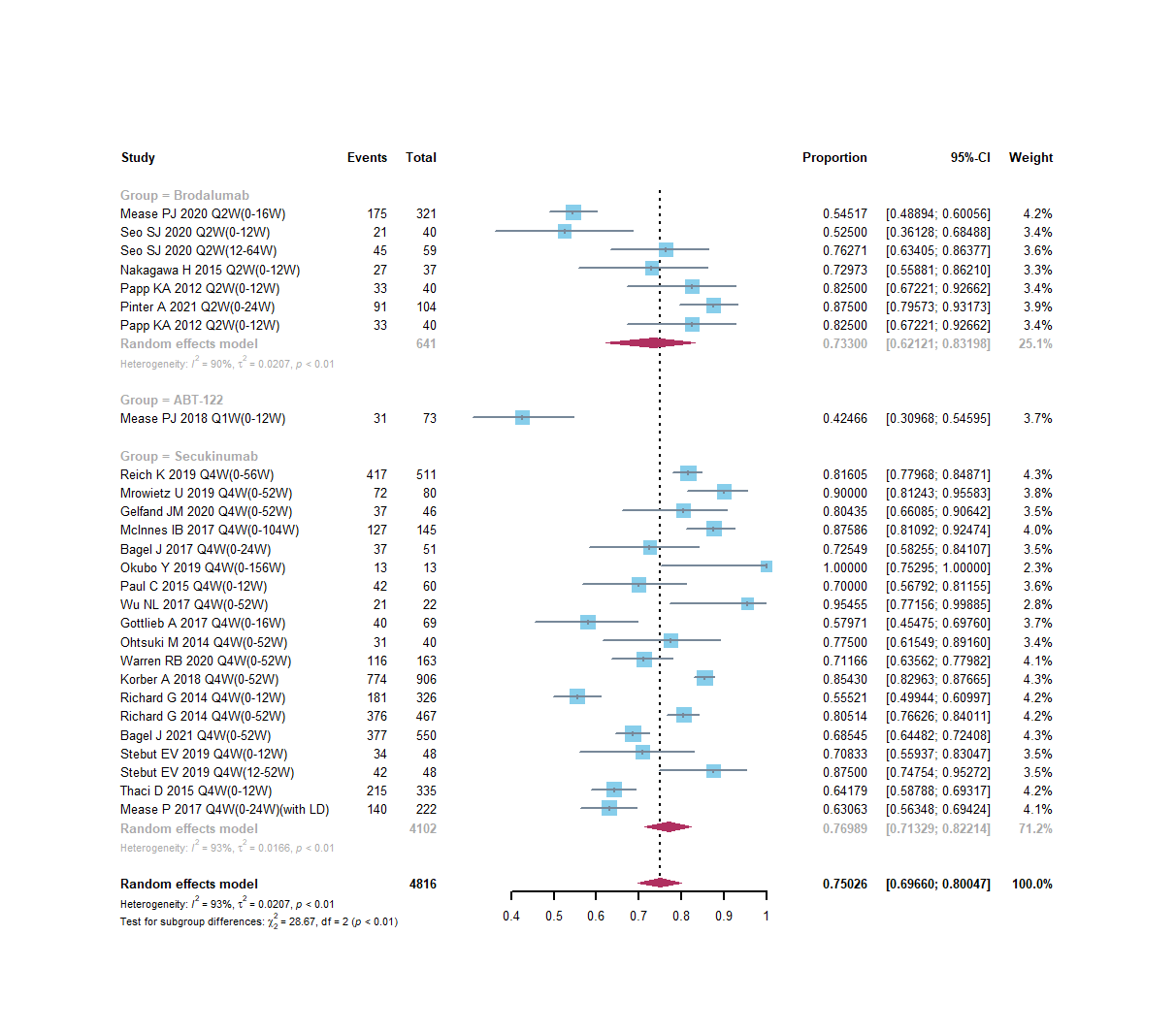

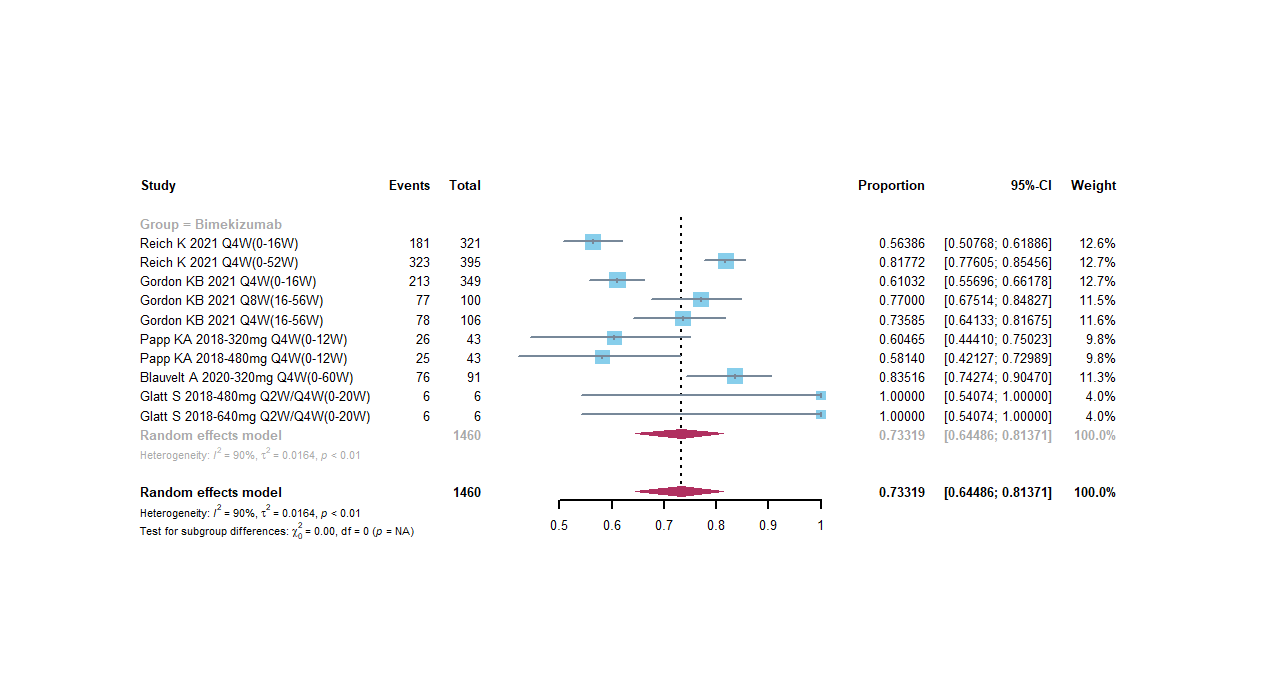


**Supplementary Figure 9.** Incidence of adverse events caused by anti-IL-17 agents over different treatment durations.


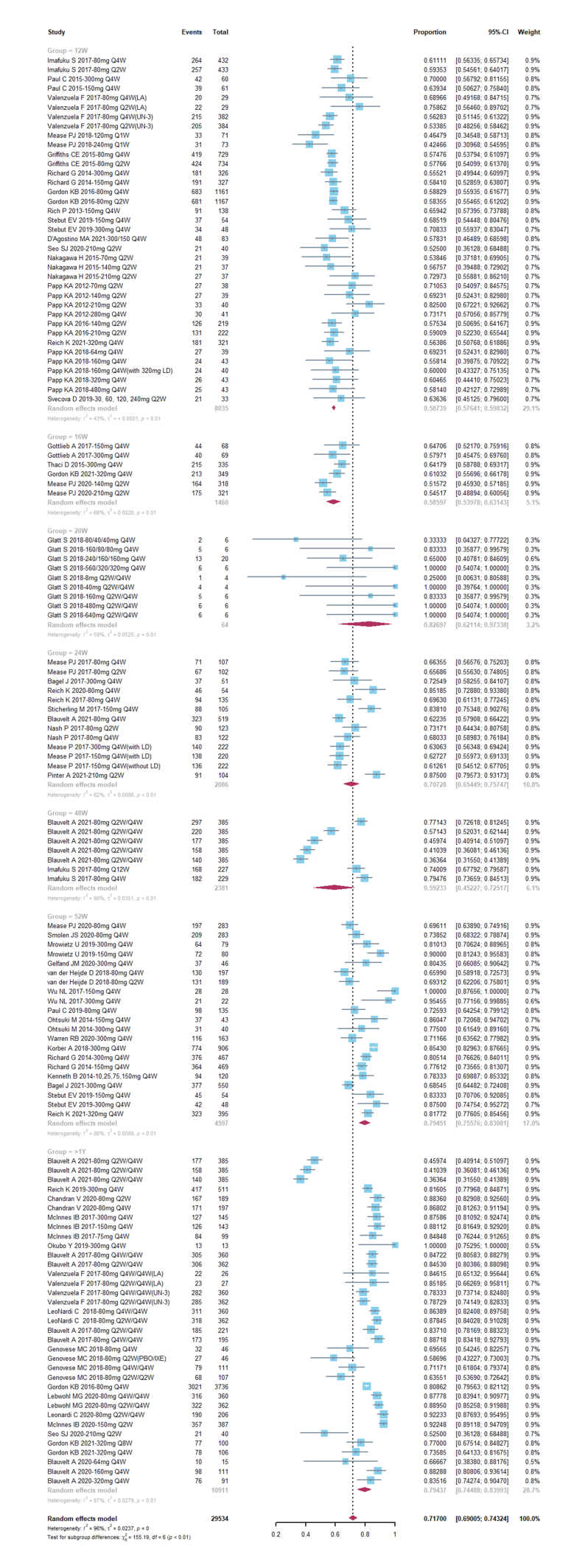


**Supplementary Figure 9.1–9.7.** Subgroup analysis of adverse events caused by anti-IL-17 drugs over different treatment durations.


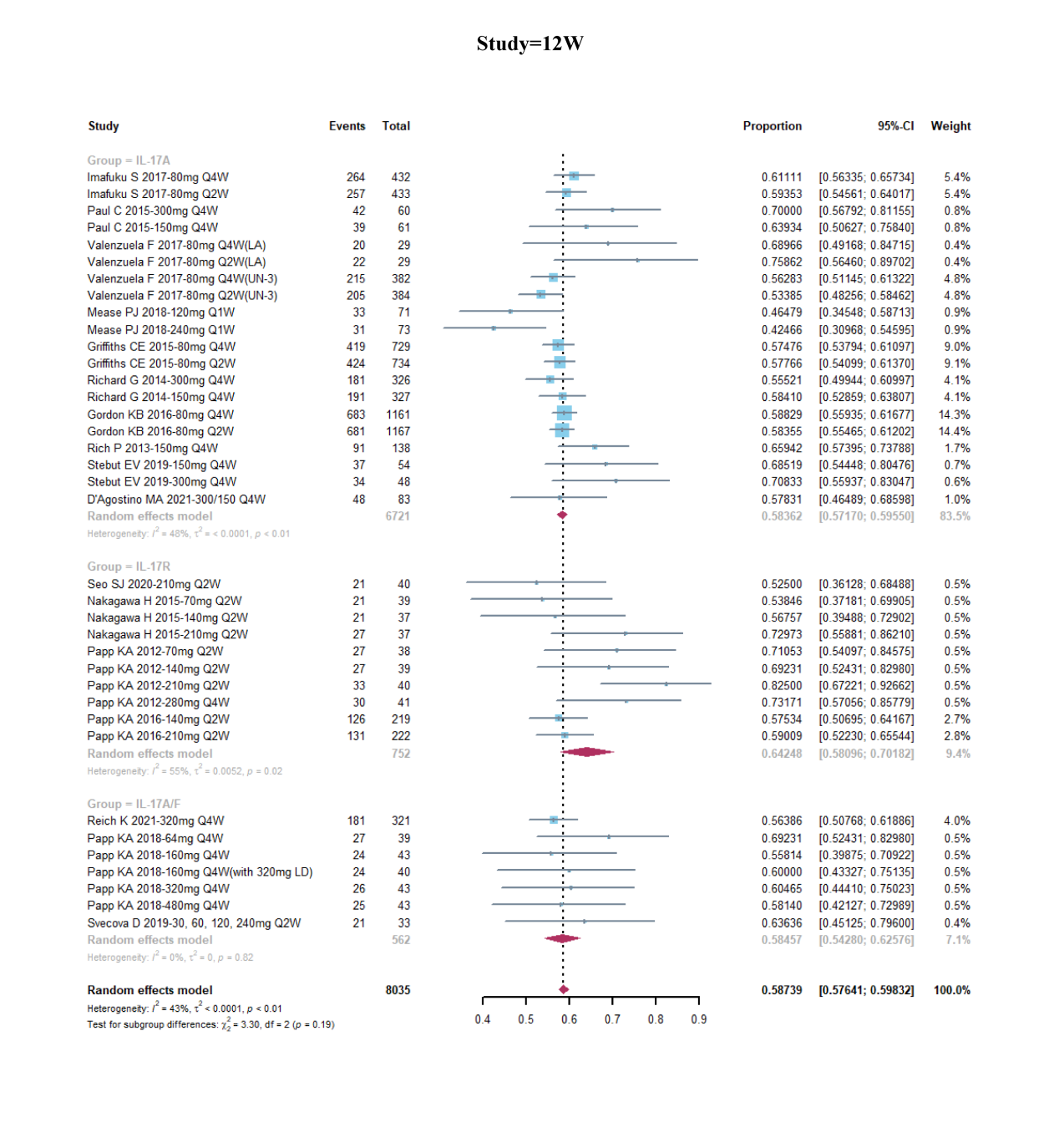

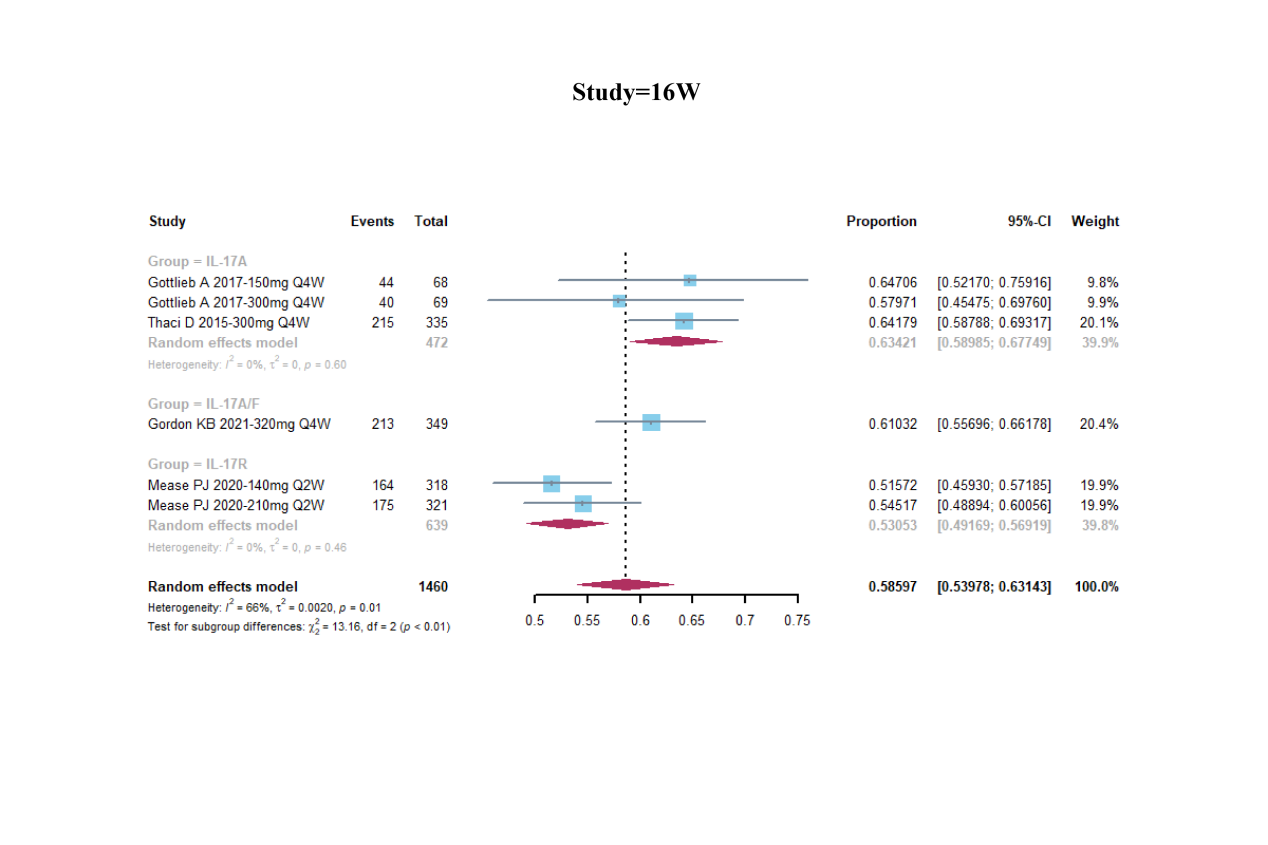

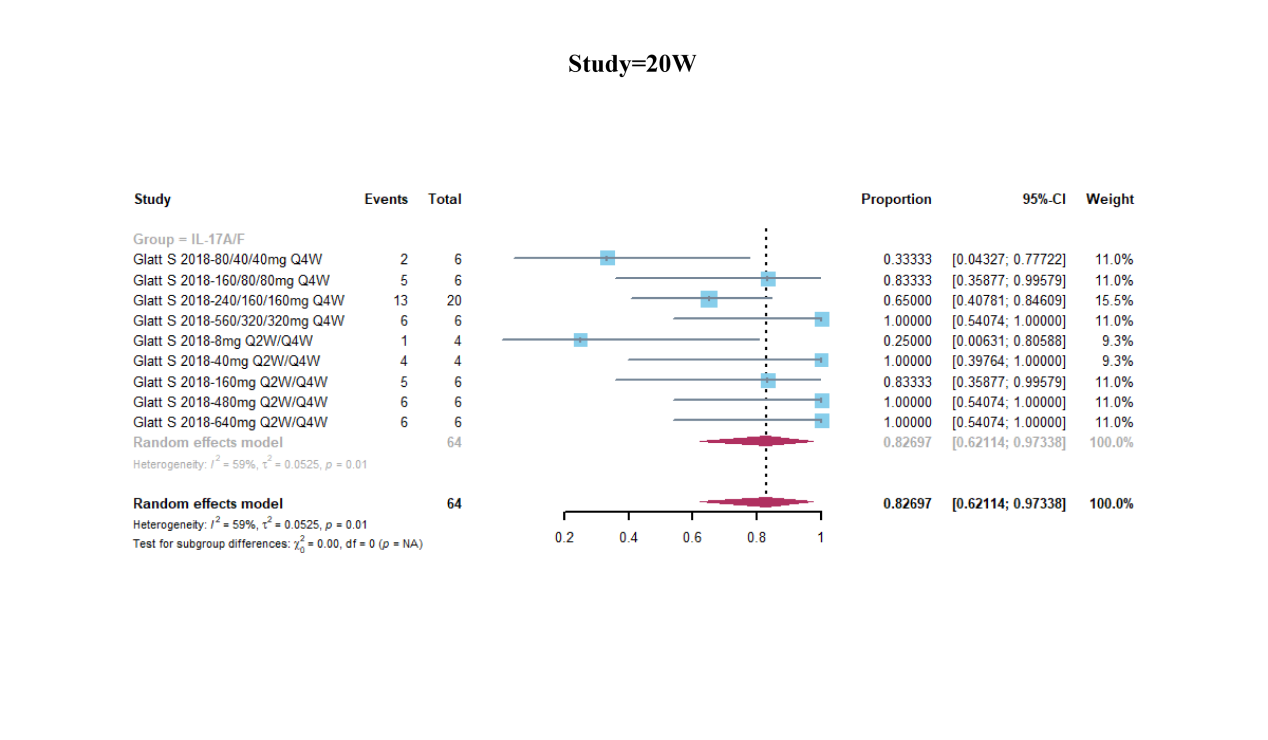

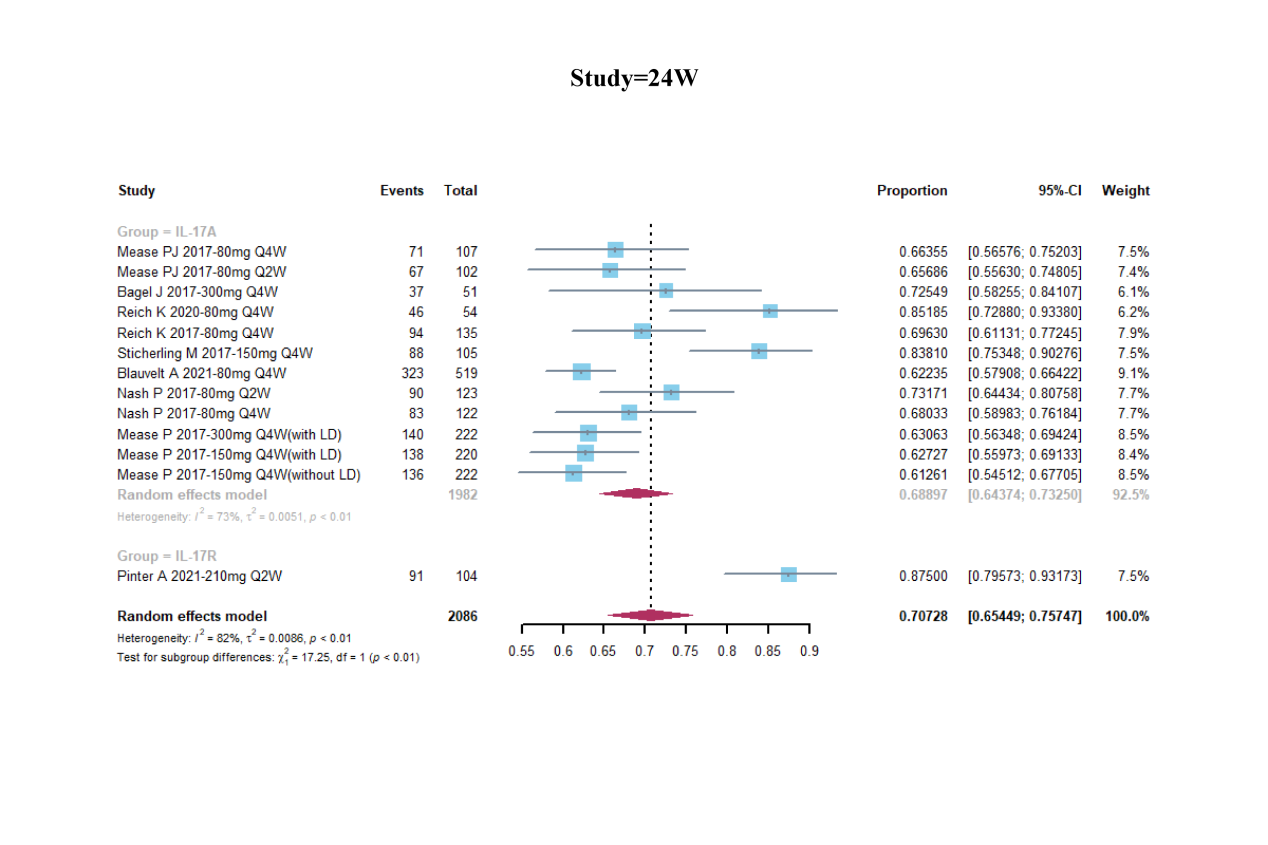

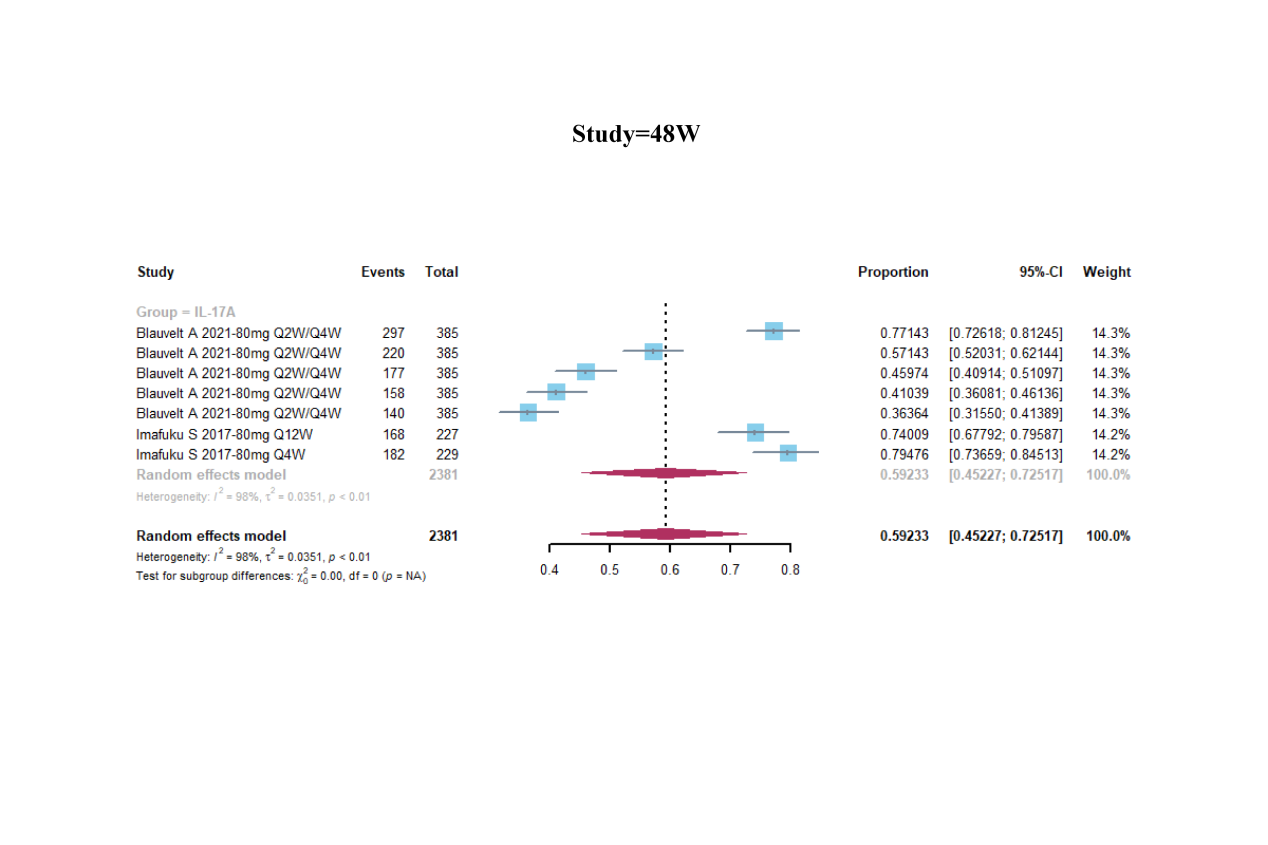

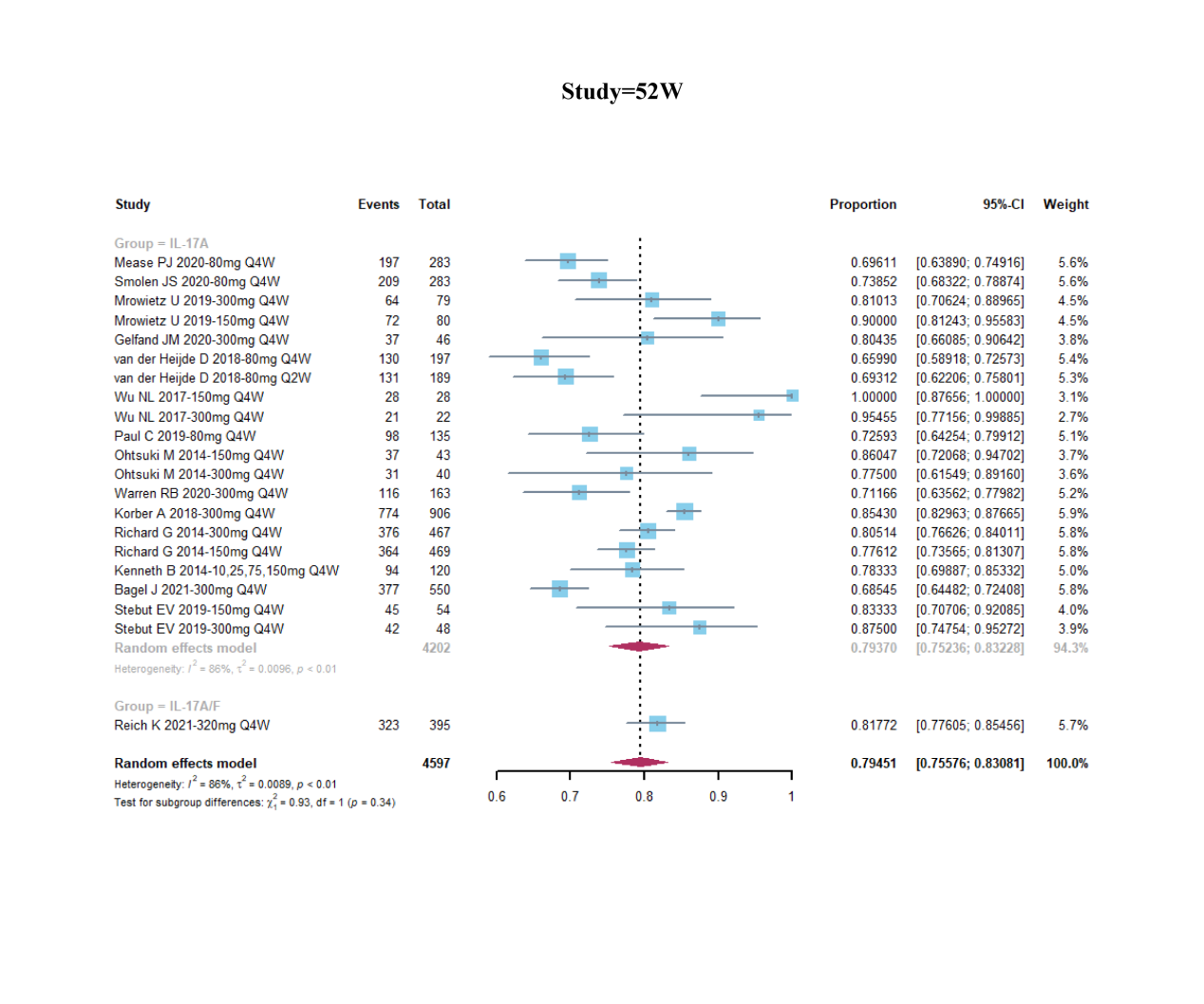

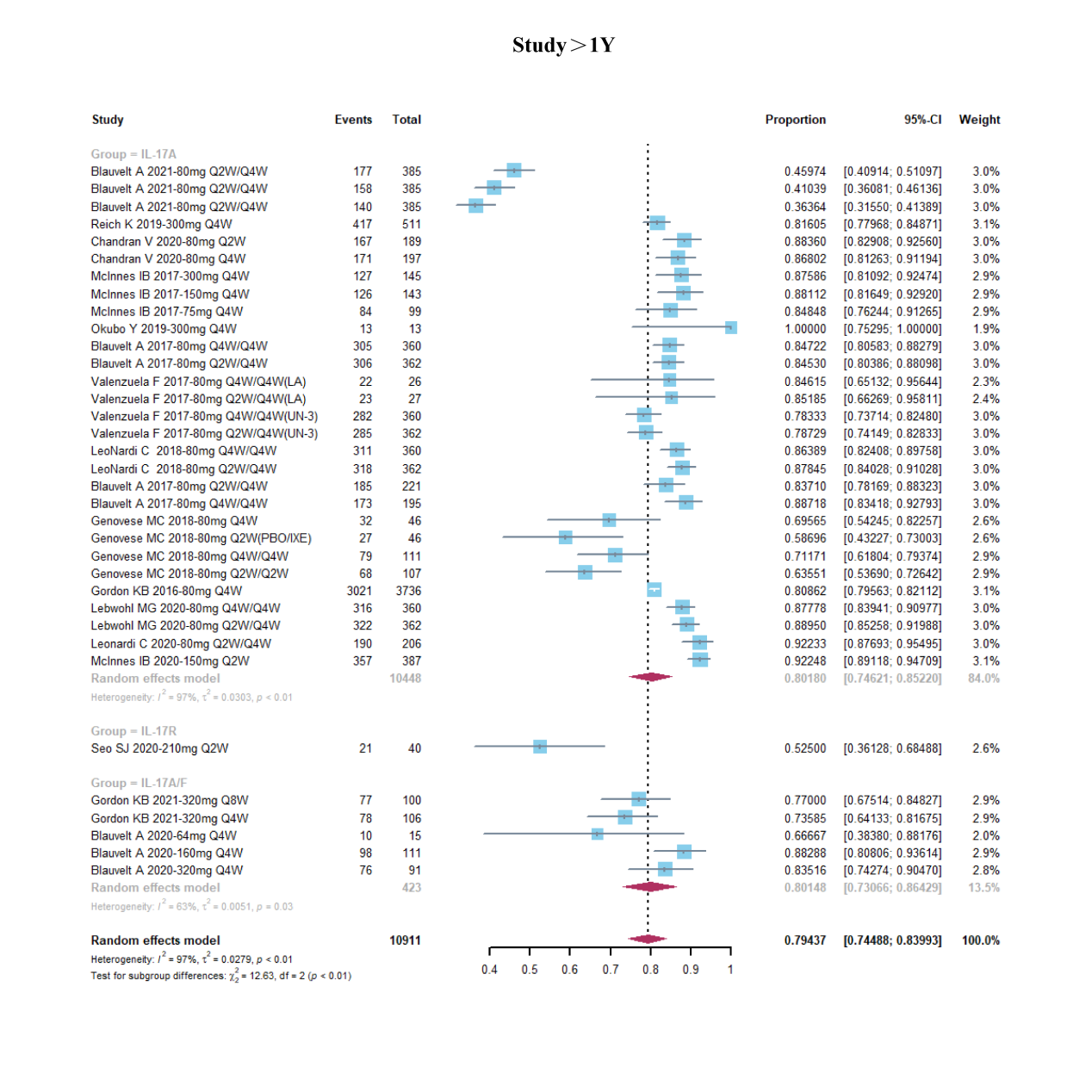

Supplement: Supplementary file 1 [file DataSheet_1.docx]
