# Supplementary material for: Adverse events associated with anti-IL-17 agents for psoriasis and psoriatic arthritis: a systematic scoping review
Source: Front Immunol. 2023 Jan 31;14:993057. doi: 10.3389/fimmu.2023.993057 (PMC9928578; doi:10.3389/fimmu.2023.993057)

**Supplementary Figure 10.** The incidence of adverse events corresponds to the interval between anti-IL-17 drug treatments.


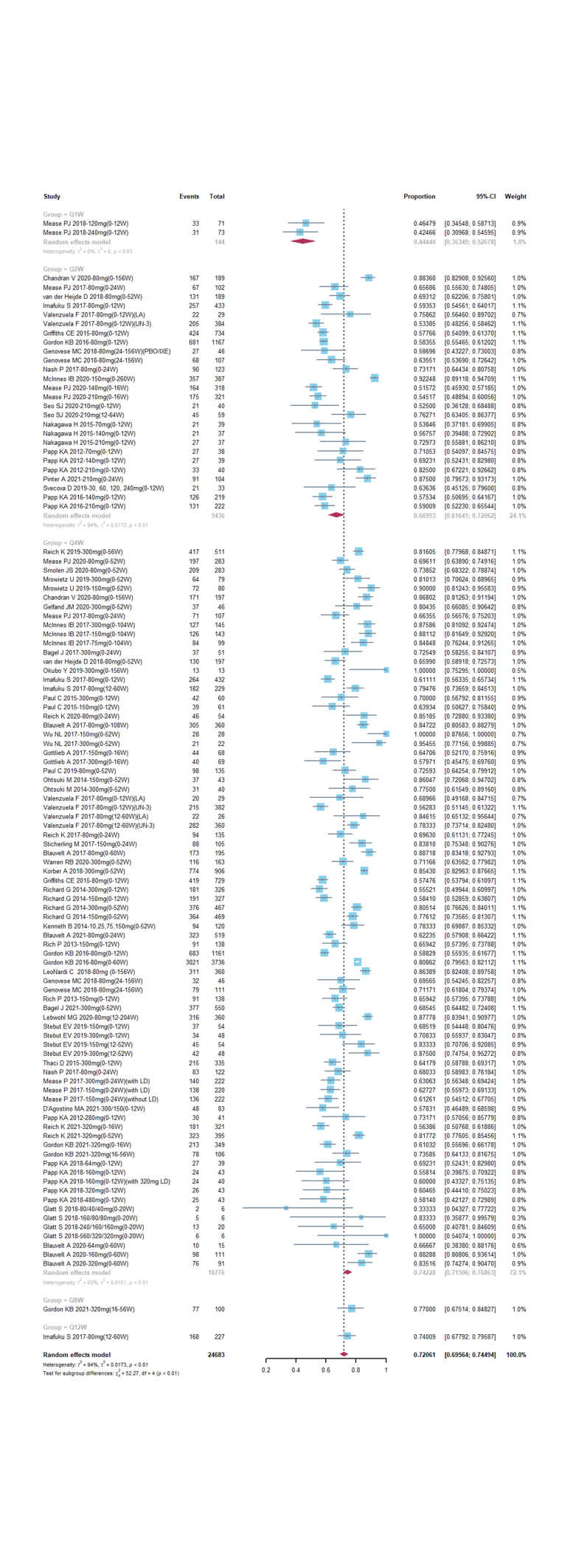


**Supplementary Figure 10.1–10.5.** Subgroup analysis of adverse events caused by anti-IL-17 drugs when administered at different intervals.


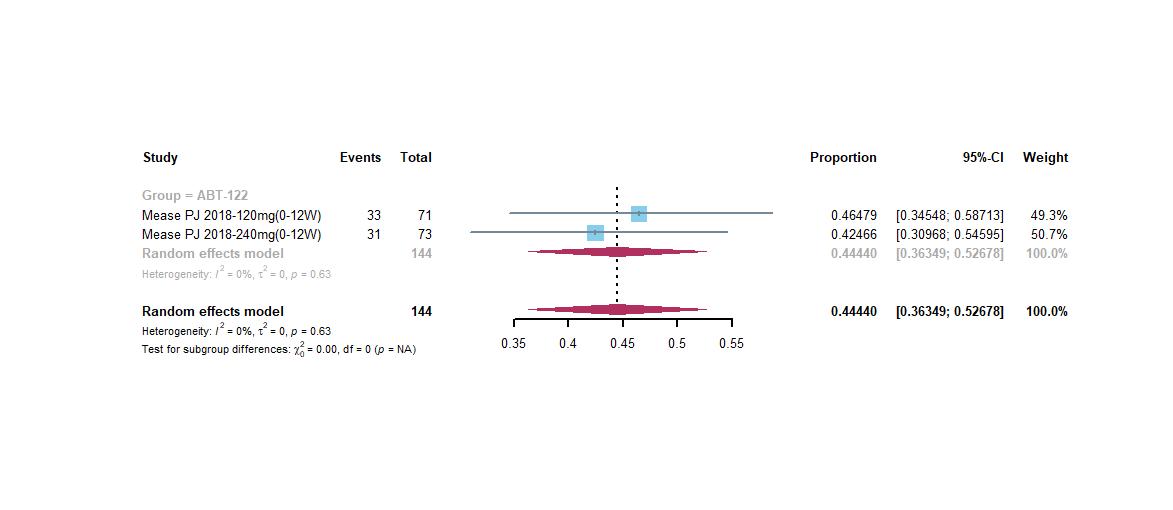

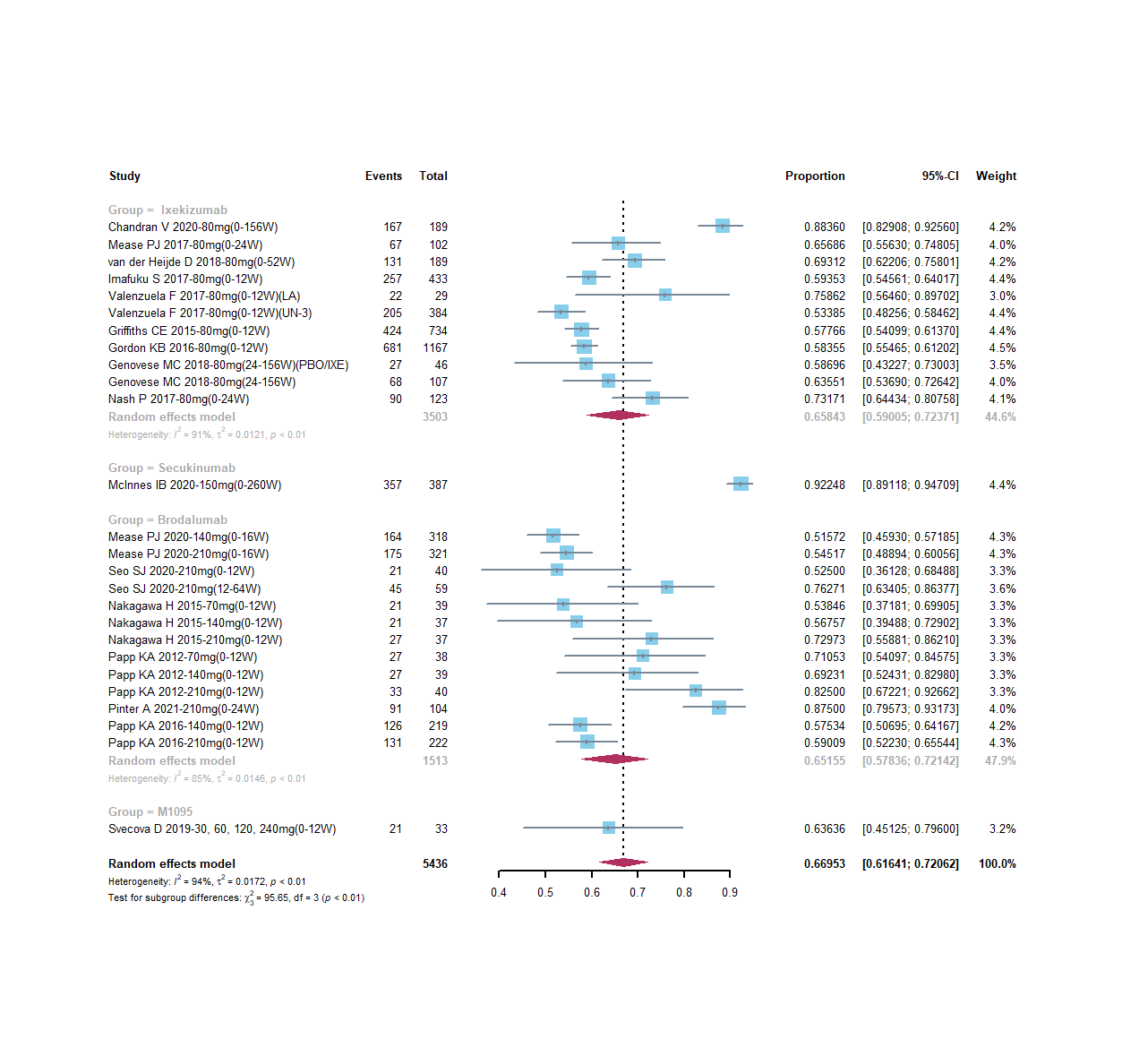

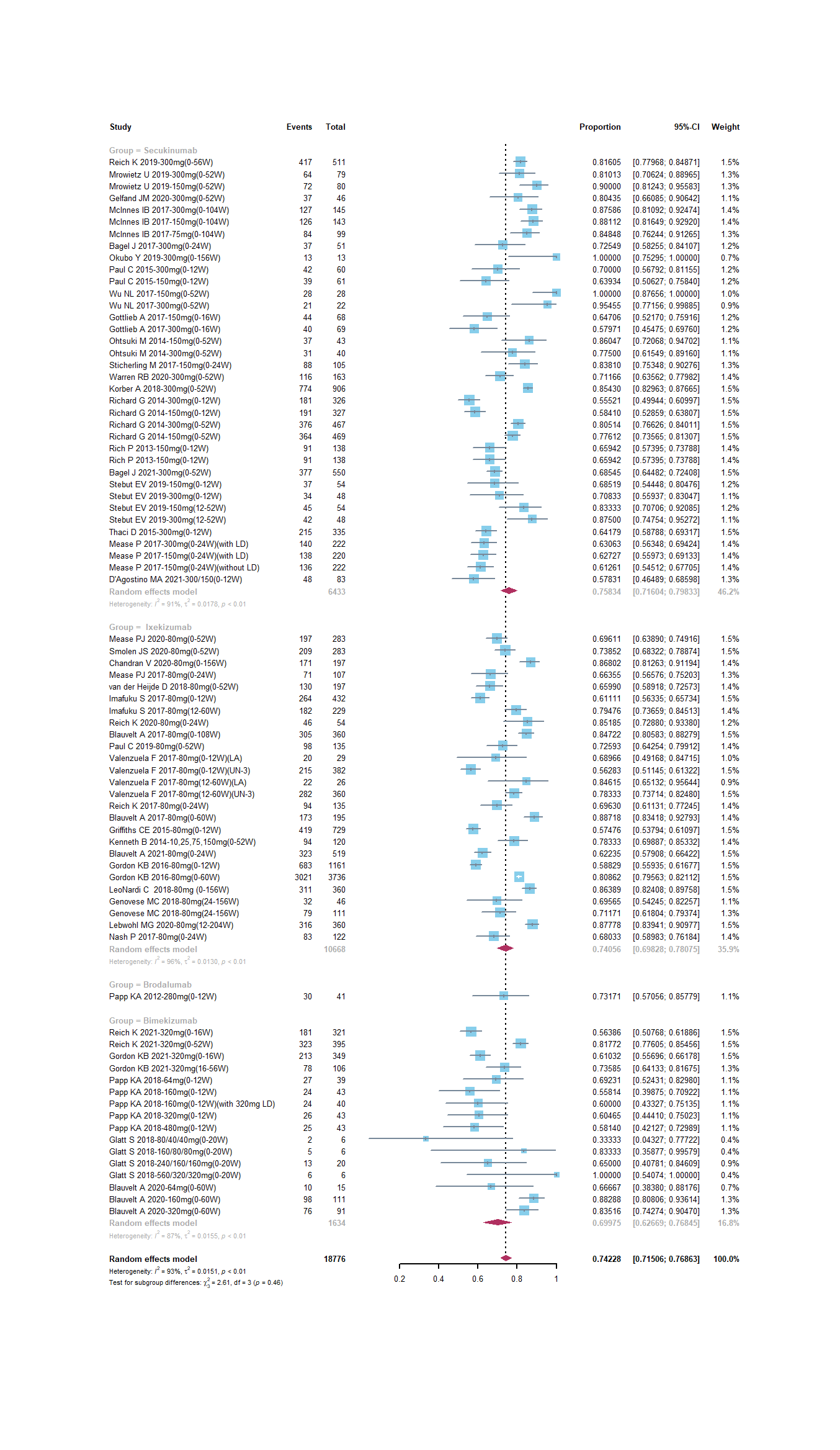

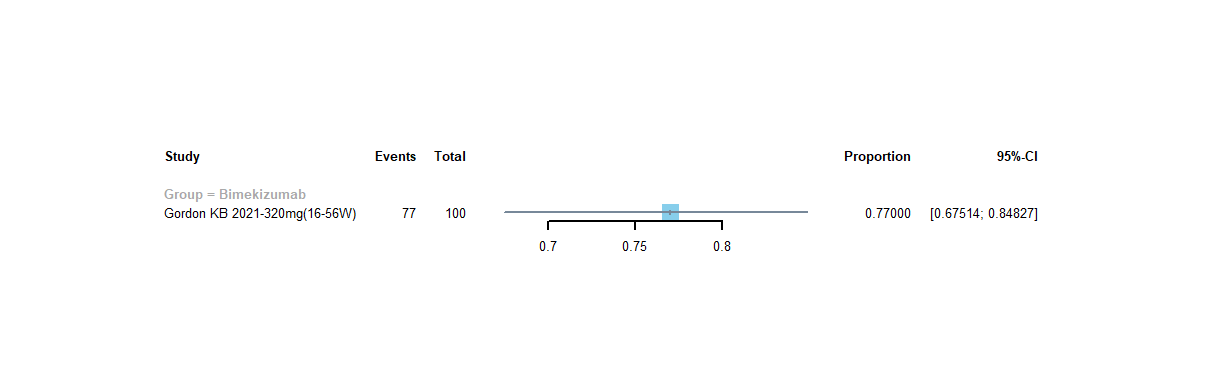

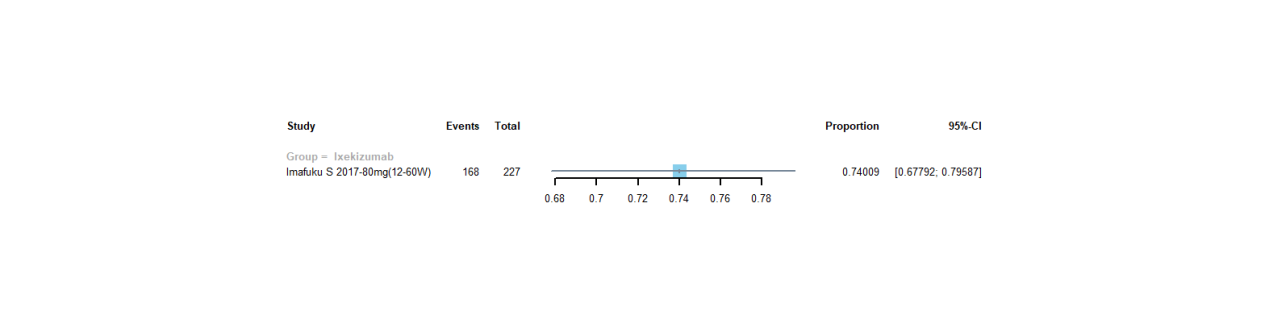


**Supplementary Figure 11.** Incidence of common adverse events caused by anti-IL-17 agents.


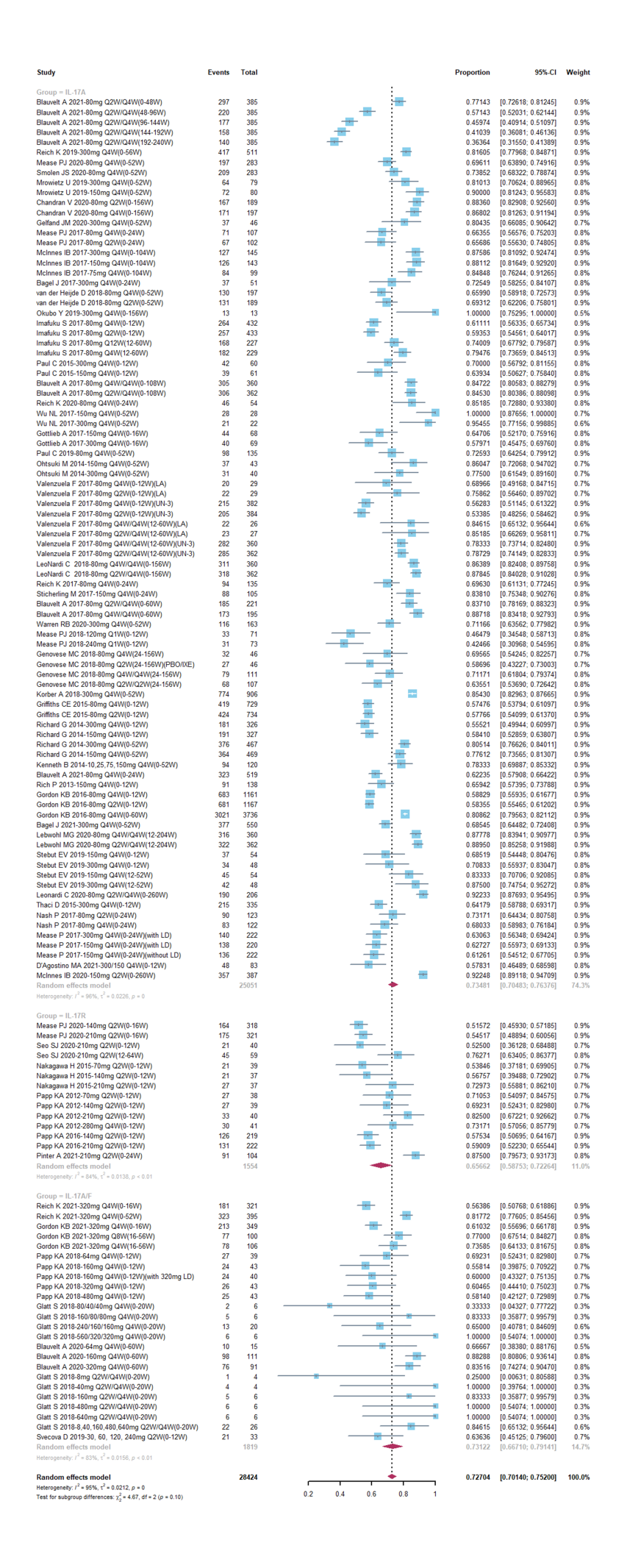


**Supplementary Figure 12.** Incidence of adverse events caused by different anti-IL-17 agents.


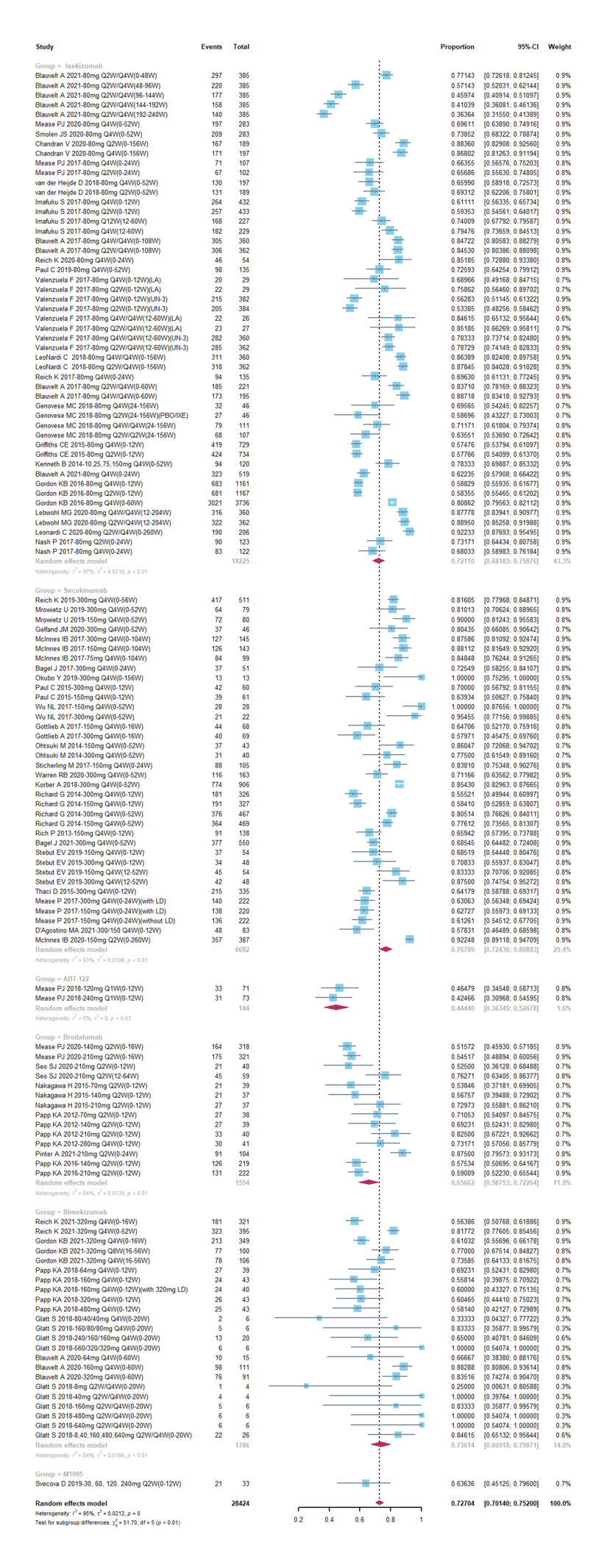


**Supplementary Figure 13.** Incidence of each type of adverse events caused by anti-IL-17 agents.


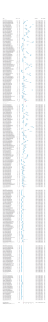


**Supplementary Figure 14.** Incidence of each type of adverse events caused by anti-IL-17 agents.


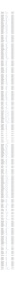


**Supplementary Figure 15.** Incidence of TEAEs caused by anti-IL-17 agents.


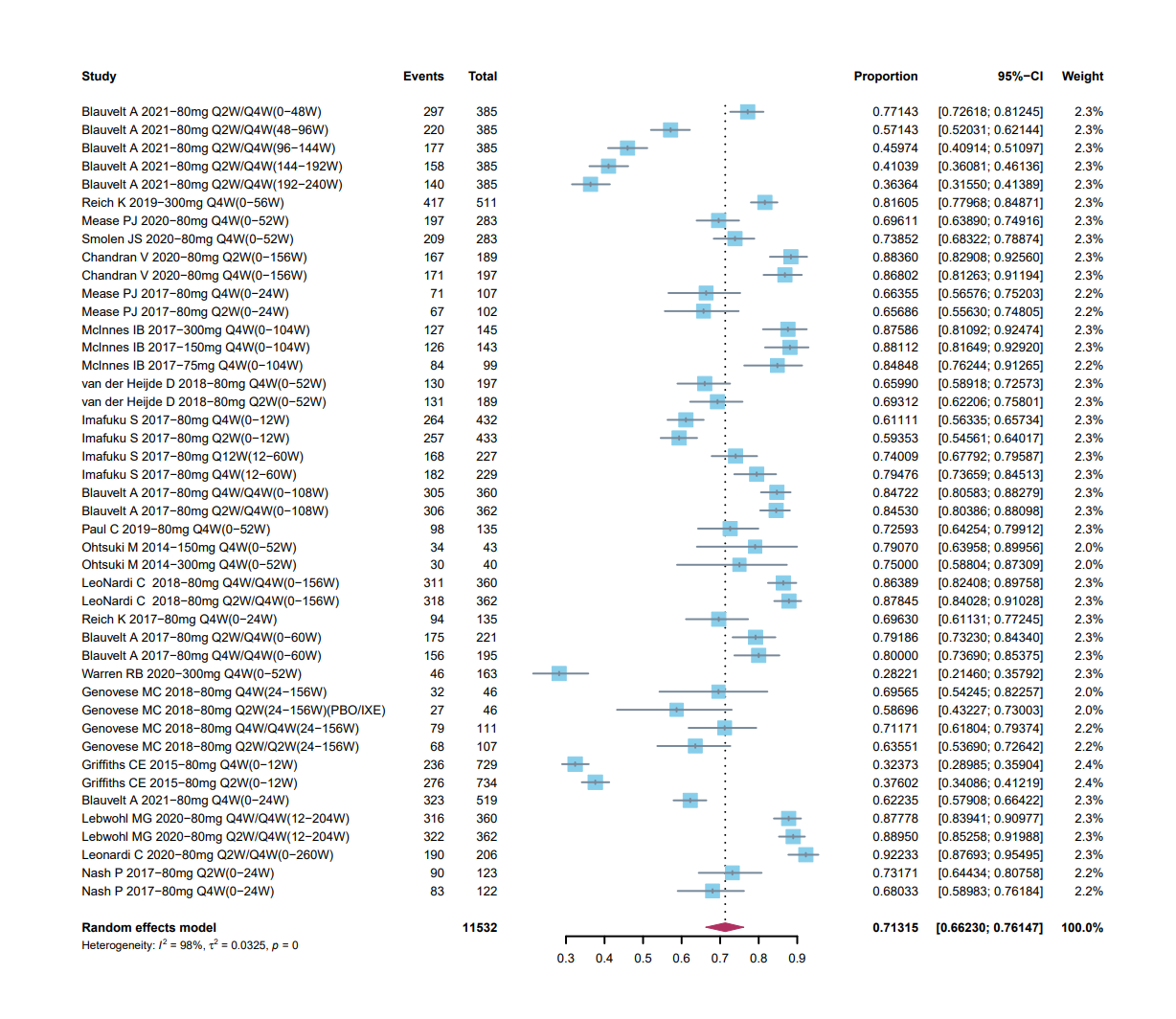


**Supplementary Figure 16.** Incidence of SAEs caused by anti-IL-17 agents.


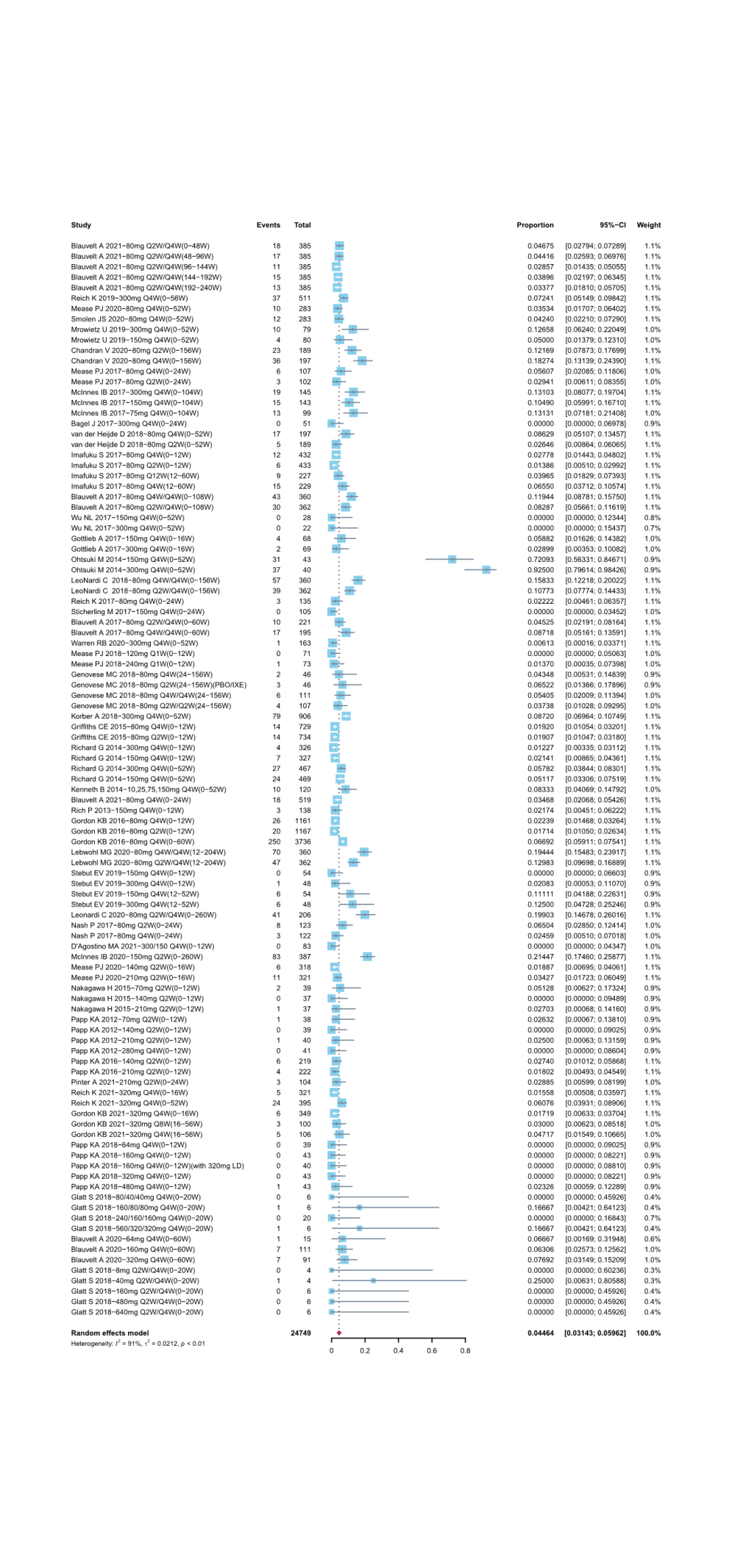

Supplement: Supplementary file 2 [file DataSheet_2.docx]
