# Supplementary material for: Adverse events associated with anti-IL-17 agents for psoriasis and psoriatic arthritis: a systematic scoping review
Source: Front Immunol. 2023 Jan 31;14:993057. doi: 10.3389/fimmu.2023.993057 (PMC9928578; doi:10.3389/fimmu.2023.993057)
Supplement: Supplementary file 4 [file Table_1.doc]

**e Table 1. Classification of adverse events of biological agents**

| **Type** | **Description** | **Performances** |
| --- | --- | --- |
| α | The cytokine release syndrome | Systemic inflammatory response: fever, fatigue, arthritis, headache, myalgia, gastrointestinal symptoms (nausea, vomiting, diarrhea), pulmonary edema, encephalopathy, etc.. |
| β | Hypersensitivity reactions | Immediate (IgE): urticaria, allergic asthma, allergic rhinitis, eosinophilia, granulocyte dysfunction, etc..  Delayed (IgG, T cell): serum sickness, vasculitis, nephritis, etc.. |
| γ | Immune/cytokine imbalance syndromes;  (chemical) reactions | Thrombocytopenia, hemolytic anemia, IgA nephropathy, herpetic dermatitis, systemic lupus erythematosus, vasculitis, thyroid disease, pernicious anemia, psoriasis, vitiligo, interstitial lung disease, multiple sclerosis, etc.. |
| δ | Cross-reactivity; (delayed) reactions | Malignant tumor, acne, etc.. |
| Σ | Non-imunological side-effects | Heart failure, psychiatric disorders, paraesthesia, hearing loss, etc.. |
