# Supplementary material for: Adverse events associated with anti-IL-17 agents for psoriasis and psoriatic arthritis: a systematic scoping review
Source: Front Immunol. 2023 Jan 31;14:993057. doi: 10.3389/fimmu.2023.993057 (PMC9928578; doi:10.3389/fimmu.2023.993057)
Supplement: Supplementary file 6 [file Table_3.doc]

**Supplementary Table 3. The Cochrane Collaboration’s tool for assessing risk of bias of included studies.**

|  | Random sequence generation | Allocation concealment | Blinding of participants and personnel | Blinding of outcome assessment | Incomplete outcome data | Selective outcome reporting | Other source of bias |
| --- | --- | --- | --- | --- | --- | --- | --- |
| Blauvelt A 2021 | + | + | + | + | + | + | ? |
| Reich K 2019 | + | + | + | + | + | + | ? |
| Mease PJ 2020 | + | + | + | + | + | + | ? |
| Smolen JS 2020 | + | ? | - | - | + | + | ? |
| Mrowietz U 2019 | + | + | + | + | + | + | ? |
| Chandran V 2020 | + | ? | + | + | + | + | ? |
| Gelfand JM 2020 | + | ? | + | + | + | + | ? |
| Mease PJ 2017 | + | + | + | + | + | + | ? |
| McInnes IB 2017 | + | ? | ? | ? | + | + | ? |
| Bagel 2017 | + | ? | ? | ? | + | + | ? |
| van der Heijde D 2018 | + | + | + | + | + | + | ? |
| Okubo Y 2019 | + | ? | + | + | + | + | ? |
| Imafuku S 2017 | + | ? | ? | ? | + | + | ? |
| Paul C 2015 | + | ? | ? | ? | + | + | ? |
| Blauvelt A 2017 | + | ? | ? | ? | + | + | ? |
| Reich K 2020 | + | ? | - | - | + | + | ? |
| Wu NL 2017 | + | ? | ? | ? | + | + | ? |
| Gottlieb A 2017 | + | ? | ? | ? | + | + | ? |
| Paul C 2019 | + | ? | + | + | + | + | ? |
| Ohtsuki M 2014 | + | ? | ? | ? | + | + | ? |
| Valenzuela F 2017 | + | - | - | - | + | + | ? |
| LeoNardi C 2018 | ? | ? | + | + | + | + | ? |
| Reich K 2017 | + | + | + | + | + | + | ? |
| Sticherling M 2017 | ? | + | - | - | + | + | ? |
| Blauvelt A 2017 | ? | ? | + | + | + | + | ? |
| Warren RB 2020 | + | + | + | + | + | + | ? |
| Mease PJ 2018 | + | + | + | + | + | + | ? |
| Genovese MC 2018 | + | + | + | + | + | + | ? |
| Korber A 2018 | ? | ? | + | + | + | + | ? |
| Griffiths CE 2015 | + | + | + | + | + | + | ? |
| Richard G 2014 | ? | ? | + | + | + | + | ? |
| Kenneth B 2014 | ? | ? | ? | ? | + | + | ? |
| Blauvelt A 2021 | + | + | + | + | + | + | ? |
| Rich P 2013 | + | + | + | + | + | + | ? |
| Gordon KB 2016 | ? | ? | + | + | + | + | ? |
| Bagel J 2021 | ? | ? | + | + | + | + | ? |
| Lebwohl MG 2020 | ? | ? | + | + | + | + | ? |
| Stebut EV 2019 | ? | ? | + | + | + | + | ? |
| Leonardi C 2020 | ? | ? | + | + | + | + | ? |
| Thaci D 2015 | + | + | + | + | + | + | ? |
| Nash P 2017 | + | + | + | + | + | + | ? |
| Mease P 2017 | ? | ? | + | + | + | + | ? |
| D'Agostino MA 2021 | + | + | + | + | + | + | ? |
| McInnes IB 2020 | ? | ? | + | + | + | + | ? |
| Mease PJ 2020 | ? | ? | + | + | + | + | ? |
| Seo SJ 2020 | + | + | + | + | + | + | ? |
| Nakagawa H 2015 | ? | ? | + | + | + | + | ? |
| Papp KA 2012 | ? | ? | + | + | + | + | ? |
| Pinter A 2021 | + | + | + | + | + | + | ? |
| Reich K 2021 | + | + | + | + | + | + | ? |
| Gordon KB 2021 | + | + | + | + | + | + | ? |
| Papp KA 2018 | + | + | + | + | + | + | ? |
| Glatt S 2018 | ? | ? | + | + | + | + | ? |
| Blauvelt A 2020 | ? | ? | + | + | ? | ? | ? |
| Glatt S 2018 | ? | ? | + | + | + | + | ? |
| Svecova D 2019 | + | + | + | + | + | + | ? |

The dark green cells (+) indicate a low risk of bias. The red cells (-) indicate a high risk of bias. The yellow cells (?) indicate an uncertain risk of bias. n/a, not applicable
