# Supplementary material for: Adverse events associated with anti-IL-17 agents for psoriasis and psoriatic arthritis: a systematic scoping review
Source: Front Immunol. 2023 Jan 31;14:993057. doi: 10.3389/fimmu.2023.993057 (PMC9928578; doi:10.3389/fimmu.2023.993057)
Supplement: Supplementary file 7 [file Table_4.docx]

| **Supplementary Table 4. Incidence of different types of AE caused by different targets** | | | | |
| --- | --- | --- | --- | --- |
| **Type** | **AE** | **IL-17A** | **IL-17A/F** | **IL-17R** |
| Type-α | Viral URI | 16.37% | 0.00% | **33.65%** |
| Type-α | URI | 8.09% | 6.72% | 8.41% |
| Type-α | Diarrhoea | 4.64% | **25.00%** | 4.61% |
| Type-α | Headache | 7.03% | 10.61% | 8.87% |
| Type-α | Back pain | 4.78% | 0.00% | 5.77% |
| Type-α | Urinary tract infection | 3.39% | 0.00% | 0.00% |
| Type-α | Candida infections | 2.89% | 10.38% | 0.00% |
| Type-α | Cough | 4.33% | 0.00% | 6.06% |
| Type-α | Arthralgia | 4.91% | 4.78% | 6.11% |
| Type-α | Nausea | 2.98% | 7.69% | 5.54% |
| Type-α | Oropharyngeal pain | 4.33% | 6.54% | 4.93% |
| Type-α | Infections | **26.98%** | 17.24% | **26.76%** |
| Type-α | Serious infections | 1.34% | 0.94% | 0.00% |
| Type-α | Fatigue | 4.18% | 0.00% | 5.91% |
| Type-α | Influenza | 6.34% | 0.00% | 1.92% |
| Type-α | Pain in extremity | 11.39% | 0.00% | 6.18% |
| Type-α | Dizziness | 2.17% | 7.69% | 0.00% |
| Type-α | Vomiting | 4.65% | 5.13% | 0.00% |
| Type-α | Abdominal pain | 0.00% | **25.00%** | 0.00% |
| Type-α | Gastrointestinal disorders | 12.96% | 13.16% | 0.00% |
| Type-α | Others | 3.31% | 4.88% | 7.51% |
| Type-β | Injection-site reaction | **9.52%** | **11.54%** | 4.58% |
| Type-β | Bronchitis | 5.58% | 6.06% | 0.00% |
| Type-β | Injection-site erythema | 4.51% | 0.00% | 0.00% |
| Type-β | Allergic reaction/hypersensitivity events | 4.26% | 0.00% | 0.00% |
| Type-β | Nasopharyngitis | **16.05%** | **12.62%** | **10.27%** |
| Type-β | Sinusitis | 4.63% | 0.00% | 0.00% |
| Type-β | Pruritus | 4.06% | **9.09%** | 0.00% |
| Type-β | Decreased neutrophils | 3.95% | 0.00% | 0.00% |
| Type-β | Eczema | 5.57% | 0.00% | 4.04% |
| Type-β | Urticaria | 9.02% | 0.00% | **8.08%** |
| Type-β | Neutropenia | 0.46% | 7.14% | 0.94% |
| Type-β | Others | 2.19% | 0.00% | 0.00% |
| Type-γ | Hypertension | **4.59%** | **4.82%** | 0.00% |
| Type-γ | Hepatic event | **3.41%** | 2.75% | 0.00% |
| Type-γ | Cerebro‐cardiovascular events | 1.55% | 0.86% | 0.00% |
| Type-γ | Cytopenia | 1.27% | 3.66% | 0.00% |
| Type-γ | PSA/PSO | 2.86% | **6.11%** | 0.00% |
| Type-γ | Ulcerative colitis | 0.38% | 0.00% | 0.00% |
| Type-γ | Crohn's disease | 0.24% | 0.00% | 0.00% |
| Type-γ | Inflammatory bowel disease | 0.24% | 0.00% | 0.00% |
| Type-γ | Others | 2.83% | 3.03% | 6.78% |
| Type-ϵ | Suicidal ideation and behaviour | 0.00% | 0.25% | 0.31% |
| Type-ϵ | Nervous system disorders | **20.37%** | **21.05%** | 0.00% |
| Type-ϵ | Depression | 1.33% | 0.00% | 4.81% |

**Abbreviation:** URI,Upper Respiratory Tract Infection; PSA, psoriatic arthritis; PSO,psoriasis.
